# Planning under uncertainty for safe robot exploration using Gaussian process prediction

**DOI:** 10.1007/s10514-024-10172-6

**Published:** 2024-08-28

**Authors:** Alex Stephens, Matthew Budd, Michal Staniaszek, Benoit Casseau, Paul Duckworth, Maurice Fallon, Nick Hawes, Bruno Lacerda

**Affiliations:** 1 Oxford Robotics Institute, University of Oxford, Oxford, UK; 2 InstaDeep, London, UK

**Keywords:** Safe exploration, Mobile robots, Markov decision processes, Gaussian processes

## Abstract

The exploration of new environments is a crucial challenge for mobile robots. This task becomes even more complex with the added requirement of ensuring safety. Here, safety refers to the robot staying in regions where the values of certain environmental conditions (such as terrain steepness or radiation levels) are within a predefined threshold. We consider two types of safe exploration problems. First, the robot has a map of its workspace, but the values of the environmental features relevant to safety are unknown beforehand and must be explored. Second, both the map and the environmental features are unknown, and the robot must build a map whilst remaining safe. Our proposed framework uses a Gaussian process to predict the value of the environmental features in unvisited regions. We then build a Markov decision process that integrates the Gaussian process predictions with the transition probabilities of the environmental model. The Markov decision process is then incorporated into an exploration algorithm that decides which new region of the environment to explore based on information value, predicted safety, and distance from the current position of the robot. We empirically evaluate the effectiveness of our framework through simulations and its application on a physical robot in an underground environment.

## Introduction

Mobile robot tasks often involve navigating through environments with hazardous conditions. These hazards can take the form of steep terrain for planetary rovers, underwater currents or shallow water depth for underwater vehicles, or radiation levels in disaster recovery or nuclear inspection. In this paper, we propose a safe exploration method that handles cases where the values of the hazardous environmental features are *unknown* a priori. We consider two settings: one where the robot already has a map of the area to be explored and wants to learn about the hazards in a safe manner, and another where the robot lacks a map and needs to create one while considering the hazards and maintaining safety.

The core of our framework is based on modelling the robot’s navigation under the hazardous features with unknown values as a partially known state Markov decision process (PKSMDP), and utilising a Gaussian process (GP) to estimate the unknown values across the environment. The GP’s capability to quantify its *prediction uncertainty* is crucial, since ensuring safety with a high degree of certainty requires the robot to assess its confidence in the predictions about the environmental features. To plan considering this uncertainty, we propose encoding the GP uncertainties into the transition probabilities of the PKSMDP, yielding a model we call the *Estimated* MDP (EstMDP).

We propose two novel exploration algorithms that use the EstMDP to plan safe paths to states expected to be informative. These safe paths take into account the probability of falling into an unsafe state when navigating to an informative state, or when travelling back from an informative state to a state with guaranteed safety. The first algorithm, *SafeEstMDP-Process*, extends the work in Turchetta et al. (2016) to handle probabilistic transition models and support more complex safety specifications. SafeEstMDP-Process selects new goal locations that reduce the uncertainty of the GP estimates of the unknown hazard. The second algorithm, *SafeEstMDP-Map*, integrates this framework with an online mapping system, thereby extending it to settings where the map of the workspace the robot is operating in is also unknown. SafeEstMDP-Map incrementally builds a navigation PKSMDP online using data from onboard sensors, and selects new goal locations based on expected information gain of the underlying occupancy map representation of the workspace.

We extensively evaluate our algorithms in simulations of real-world environments, empirically showing that it significantly outperforms the work of Turchetta et al. (2016) and hand-crafted baselines, safely achieving increased environmental exploration while also minimising the distance travelled in the process. We also report on a trial performed using a Boston Dynamics Spot deployed in an underground mine section at Corsham, Wiltshire, UK.

The contributions of this paper are as follows:

- The PKSMDP, a novel model which formalises problems of safe exploration with unknown hazardous features under uncertain action outcomes;
- The encoding of GP predictions into the PKSMDP transition function, yielding the EstMDP planning model;
- Two exploration algorithms, under different assumptions of prior knowledge of the environment map, which make use of the EstMDP to decide where to explore next;
- An empirical evaluation emphasising the benefits of our approach based on GP predictions and planning under uncertainty.

## Related work

**Planning and exploration with MDPs and GPs.**  MDPs are a widely used formalism for planning under uncertainty for robots, e.g. Feyzabadi and Carpin (2017), Lacerda et al. (2019), Gopalan et al. (2017). GP-modelled unknown process exploration has commonly been investigated in the Bayesian optimisation setting. Sui et al. (2015) introduced the idea of using GP regression to maximise an objective function value while ensuring high probability of not sampling function values that break a safety bound. This is done by considering the upper confidence bounds given by the GP when exploring. This work has been extended to consider safety functions which are separate from the objective function (Sui et al., 2018) and to reason explicitly about the information gain about the safety of the parameters (Bottero et al., 2022), in order to improve sample efficiency.

Safe exploration of an MDP introduces the need to reason about state reachability, which is not required in the Bayesian optimisation setting where observations are assumed to be taken freely across the domain. An early work on safe exploration of MDPs was Moldovan and Abbeel (2012), which investigated the problem of exploring an MDP whilst ensuring returnability to the initial state but assumes full knowledge of the safety of states. Turchetta et al. (2016) introduced the *SafeMDP* algorithm for safe exploration of MDPs using GPs. SafeMDP reasons about both *reachability* from and *returnability* to the already visited set of states, which must be safe if the safety bound has not been broken. However, SafeMDP only considers new states to visit that are a single step from an already explored state. We extend the reachability and returnability concepts to reason about multiple-step safety probabilities along paths. Along with the inclusion of transition costs in the exploration objective, this makes our algorithm far more cost-efficient and less myopic when exploring. Finally, we alleviate SafeMDP’s deterministic transition function assumption and explore MDPs with probabilistic transitions than allow for modelling the impact of the unknown process on the robot’s dynamics. For example, if the unknown process represents underwater currents acting on an autonomous underwater vehicle (AUV), high magnitude currents could push the robot into waypoints other than the intended goal waypoint (Budd et al., 2022). Some actions may even be entirely infeasible because the water current velocity from the goal waypoint towards the AUV is higher than the maximum water-frame speed of the AUV. Wind can have similar effects on an unmanned aerial vehicle (Badings et al., 2022). If the unknown process is terrain steepness, higher terrain gradients could make it more likely that a wheeled robot slips into an unintended waypoint while trying to navigate to the goal waypoint (Rutherford et al., 2021). Other extensions of SafeMDP include multi-agent safe exploration (Zhu et al., 2020).

The standard MDP formulation assumes a fully known model of the environment, and full observability of the current state. For sequential decision-making under state uncertainty, the partially observable MDP (POMDP) formulation is commonly used (Kaelbling et al., 1998; Spaan et al., 2015; Lauri et al., 2019). Outside of the safety-constrained setting, GPs have been used as POMDP belief models to carry out unknown process exploration in GP-modelled environments (Marchant et al., 2014; Morere et al., 2017; Flaspohler et al., 2019).

As with existing MDP safe exploration algorithms above, we do not use a partially observable formulation of the problem to avoid the computational complexity of POMDP planning. Although our approach will act more myopically than a POMDP formulation, it requires far less computation. Furthermore, given that models for exploration tend to be inaccurate, it is common to use a next-best-view approach as we propose here, rather than spend computational resources performing lookaheads based on an inaccurate model. Our approach also has similarities to a mixed observability MDP (Ong et al., 2010), where the robot’s location is observable but the environment process is only partially observable.

**Exploration of unknown environments.**  The general problem of exploration of unknown environments has been addressed through frontier-based approaches, which aim to guide the robot towards unknown areas of the map (Yamauchi, 1998; Yamauchi et al., 1998; Freda & Oriolo, 2005). The research on this topic has recently received a lot of attention due to the DARPA Subterranean Challenge (SubT), where teams of robots must autonomously explore underground environments. In the context of SubT, extensive teams of researchers have developed and tested complex integrated systems which address problems such as localisation and mapping in GPS-denied environments, navigation in adverse terrains, or coordination of heterogeneous multi-robot teams under communication constraints (Tranzatto et al., 2022; Rouček et al., 2022; Hudson et al., 2022; Morrell et al., 2022; Scherer et al., 2022). Numerous exploration algorithms have been developed in the context of SubT, a significant portion of which rely on frontier-based methods (Bayer & Faigl, 2019; Dang et al., 2020; Williams et al., 2020). Our SafeEstMDP-Map approach also uses a notion of frontiers to drive the robot towards unknown areas of the map. However, because it must maintain safety with regards to the unknown process, we also consider possible points which are not at the edge of known space but for which the GP prediction of the unknown process still has high variance. Given the large scale of the SubT domains, exploration approaches are often hierarchical, with the aim of ensuring thorough exploration at a local level whilst considering when to move to other unexplored areas of the map at a global level (Dang et al., 2020; Cao et al., 2021; Kim et al., 2021). Furthermore, approaches used in SubT have a strong focus on the *traversability* of edges in the navigation graph. In contrast, we either assume the traversability is given, in the form of a navigation MDP, or that traversability can be checked by simple ray casting, assuming that the robot can traverse an edge unless there is an obstacle on the way. Moreover, we do not separate the exploration algorithm between local and global levels. This is done because our focus is on the use of GPs to model an external unknown process and reason about safety with regards to it. Note that this aspect goes beyond what is considered in the SubT works. We also note that our approach can in principle be integrated with these more complex exploration approaches, ensuring an extra layer of safety against external processes.

## Preliminaries

### Markov decision processes & constrained reachability

#### Definition 1

An MDP is defined as a tuple $\mathcal {M}=\langle S, \overline{s},A, T, c\rangle$, where *S* is a finite set of states; $\overline{s} \in S$ is the initial state; *A* is a finite set of actions; $T:S \times A \times S \rightarrow [0,1]$ is a probabilistic transition function; and $c:S\times A \rightarrow \mathbb {R}_{\ge 0}$ is a cost function.

Examples of cost functions are the expected time to execute an action, or the expected energy required to do so. A *path* through an MDP is a sequence $w=s_0 {\mathop {\rightarrow }\limits ^{a_0}}s_1{\mathop {\rightarrow }\limits ^{a_1}}...$ where $T(s_i,a_i,s_{i+1})>0$ for all $i \in \mathbb {N}$. We denote the set of all paths of $\mathcal {M}$ starting from state *s* as $\textit{Path}_{\mathcal {M},s}$. The choice of action to take at each step of the execution of an MDP is made by a *policy*. In this paper, we consider *deterministic, stationary* policies, defined as functions $\pi : S \rightarrow A$ that map each state $s \in S$ to the action to execute in *s*, and denote the set of all such policies as $\Pi$. Given an MDP $\mathcal {M}$ and a policy $\pi \in \Pi$, we can define a probability measure $\textit{Pr}_{\mathcal {M},s}^\pi$ over the set of paths $\textit{Path}_{\mathcal {M},s}$ (Kemeny et al., 1976). Furthermore, for a measurable function $X:\textit{Path}_{\mathcal {M},s}\rightarrow \mathbb {R}$, we write $\textit{E}_{\mathcal {M},s}^{\pi }(X)$ to denote the expected value of *X* with respect to $\textit{Pr}_{\mathcal {M},s}^{\pi }$.

We consider *cost-optimal reach-avoid* problems for which the probability of satisfaction might be less than one. These involve identifying a policy to reach a set of goal states whilst avoiding a set of forbidden states.

#### Definition 2

Let $G \subset S$ be a set of goal states and $F \subset S$ be a set of forbidden states. We define the set of paths that reach *G* whilst avoiding *F* as:

1 $$\begin{aligned} {\textit{reach}_{\lnot {F},{G}}} =&\{(s_0{\mathop {\rightarrow }\limits ^{a_0}}s_1{\mathop {\rightarrow }\limits ^{a_1}} \ldots ) \in \textit{Path}_{\mathcal {M},s_0} \mid \nonumber \\&\text { exists } i \in \mathbb {N} \text { such that } \nonumber \\&s_i \in G \text { and } s_j \not \in F \text { for all } j \le i\}. \end{aligned}$$

We will consider policies that are cost-optimal, in the sense that they minimise the expected cumulative cost to reach either a goal state, or a state where reaching the goal is not possible.

#### Definition 3

Let $w= s_0{\mathop {\rightarrow }\limits ^{a_0}}s_1{\mathop {\rightarrow }\limits ^{a_1}}... \in \textit{Path}_{\mathcal {M}, s_0}$. We define $l_w$ as the timestep until which cost will be accumulated for path *w*:

2 $$\begin{aligned} l_w = \left\{ \begin{array}{ll} \min _l \text { s. t. } s_l \in G & \text {if } w \in {\textit{reach}_{\lnot {F},{G}}}\\ \min _l \text { s. t. } \  & \\ \textit{Pr}_{\mathcal {M}, s_l}^{\max }({\textit{reach}_{\lnot {F},{G}}}) = 0 & \text {otherwise}, \end{array} \right. \end{aligned}$$

where $\textit{Pr}_{\mathcal {M}, s_l}^{\max }({\textit{reach}_{\lnot {F},{G}}})$ denotes the maximum over $\Pi$ of $\textit{Pr}_{\mathcal {M}, s_l}^{\pi }({\textit{reach}_{\lnot {F},{G}}})$.

Note that the second condition in the definition of $$l_w $$ encompasses the case where a forbidden state is visited before a goal state, and the case where the path can never reach a goal state. We can now define the cost-optimal reach-avoid problem.

#### Problem 1

Let ${\textit{cumul}_{\lnot {F},{G}}}:\textit{Path}_{\mathcal {M}, s} \rightarrow \mathbb {R}_{\ge 0}$ be a function that maps a path $w=s_0{\mathop {\rightarrow }\limits ^{a_0}}s_1{\mathop {\rightarrow }\limits ^{a_1}}\dots$ to the cost accumulated up to $l_w$:

3 $$\begin{aligned} {\textit{cumul}_{\lnot {F},{G}}}(w) = \sum _{i=0}^{l_w-1} c(s_i,a_i), \end{aligned}$$

and $\Pi ^*= \{\pi \in \Pi \mid \pi = {{\,\mathrm{arg\,max}\,}}_{\pi ^{\prime }} \textit{Pr}_{\mathcal {M}, \overline{s}}^{\pi ^{\prime }}({\textit{reach}_{\lnot {F},{G}}}) \}$ be the set of policies that maximise the probability of reaching *G* whilst avoiding *F*. The cost-optimal reach-avoid problem is defined as finding the policy ${\pi _{\lnot {F},{G}}} \in \Pi ^*$ that has minimal expected cumulative cost:

4 $$\begin{aligned} {\pi _{\lnot {F},{ G}}} = {\mathop {\mathrm{arg\,min}}\limits _{\pi \in \Pi ^*}}\, \textit{E}_{\mathcal {M},\overline{s}}^\pi ({\textit{cumul}_{\lnot {F},{G}}}). \end{aligned}$$

The above optimisation problem is a variant of a safest and stochastic shortest path problem (Teichteil-Königsbuch 2012). The minimal expected cost for these problems is known to converge to a finite value. We will write $\textit{E}_{\mathcal {M},\overline{s}}^*({\textit{cumul}_{\lnot {F},{G}}})$ to denote the (minimal) expected value corresponding to ${\pi _{\lnot {F},{ G}}}$. In order to find ${\pi _{\lnot {F},{G}}}$, we encode the constrained reachability problem in *co-safe linear temporal logic* and use the approach presented in Lacerda et al. (2019).

### Gaussian processes

A GP (Rasmussen & Williams, 2006) is defined as a collection of random variables, any finite number of which have a joint Gaussian distribution. A GP is fully specified by its mean function *m*(*s*) and kernel function  $k(s, s^{\prime })$, i.e. $f(s) \sim \mathcal{G}\mathcal{P}(m(s), k(s, s^{\prime }))$. We let $m(s) = 0$ without loss of generality. The kernel function *k* is parameterised by hyperparameters $\theta$ that encode prior assumptions over the unknown function *f*. We make the standard modelling assumptions that *f* has bounded norm in the Reproducing Kernel Hilbert Space associated with the chosen kernel function, and also that it is Lipschitz continuous with respect to some metric $d(\cdot , \cdot )$ on *S* (Turchetta et al., 2016; Wachi et al., 2018).

#### Definition 4

Let $\mathcal {D}=\{ s_i, z_i \}^{n_{\mathcal {D}}}_{i=1}$ be a dataset of $n_{\mathcal {D}}$ noisy observations of the form $z_i=f(s_i)+\epsilon _i$, where each $\epsilon _i \sim \mathcal {N}(0, \sigma ^2)$. For a test point $s_*$, the GP posterior is defined as:

5 $$\begin{aligned}&P^{\mathcal{G}\mathcal{P}}(f(s_*) \mid \mathcal {D}) \sim \mathcal {N}({\textbf {k}}_*^\textsf{T}({\textbf {K}} + \sigma ^2{\textbf {I}})^{-1} {\textbf {z}}, \nonumber \\&\quad k(s_*,s_*)-{\textbf {k}}_*^\textsf{T}({\textbf {K}} + \sigma ^2{\textbf {I}})^{-1} {\textbf {k}}_*), \end{aligned}$$

where ${\textbf {z}} = [z_1, \dots , z_{{n_d}}]^\textsf{T}$; ${\textbf {I}} \in \mathbb {R}^{n_\mathcal {D} \times n_\mathcal {D}}$ is the identity matrix; ${\textbf {K}} \in \mathbb {R}^{n_\mathcal {D} \times n_\mathcal {D}}$ is the positive semi-definite kernel matrix such that ${\textbf {K}}_{i,j}=k(s_i,s_j)$; and ${\textbf {k}}_{*}=[k(s_1,s_*), \ldots k(s_{{n_d}},s_*)]^\textsf{T}$.

It is possible to account for noise in the dataset GP inputs $s_i$ as well as observations $z_i$ of $f(s_i)$ by transforming the GP to maintain the noise-free input assumption. This is done by adding a correction term to the GP observation noise (McHutchon & Rasmussen, 2011).

### Occupancy map models of unknown environments

Consider a bounded environment $X \subset \mathbb {R}^n$, partitioned into free and occupied space. This is represented by sets $X_{\textit{free}}$ and $X_{\textit{occ}}$, respectively. We will maintain these sets using *occupancy maps* (Elfes, 1989) as they provide a versatile representation of unstructured environments observed through noisy sensor measurements. To simplify the presentation, we will consider $n=2$ for the remainder of the paper, i.e., we assume the robot is deployed in an environment which can be navigated in a horizontal 2D plane. However, the methods proposed here can generalise to consider a 3D environment. Thus, we represent the environment as a grid map $\mathbb {M}=\{m_1, \ldots m_\ell \}$ of $\ell$ cells $m \subset \mathbb {R}^2$.

We keep a classification of cells $m \in \mathbb {M}$ as either free space, occupied space, or unknown space. There are several ways to maintain such a classification. In our implementation, we use the OctoMap framework (Hornung et al., 2013), an octree-based library that provides a probabilistic occupancy estimate of each 3D voxel in the environment, projecting it to 2D by taking a slice of the OctoMap at the height of the robot’s lidar.

We are interested in maximising exploration coverage over an unknown environment. To do so efficiently, we use the notion of *volumetric gain* from Dang et al. (2020), which estimates the unmapped volume that could be perceived by an onboard sensor from a given location $x \in X$.

#### Definition 5

Let $N^{x}$ be the set of cells observed from location $x \in X$. We define volumetric gain at *x* as a weighted sum over the cells in each class observable from *x*, by casting uniformly-spaced rays outwards in all directions:

6 $$\begin{aligned} \texttt {gain}(x) = {\alpha }_{\textit{unk}} N^{x}_{\textit{unk}} + \alpha _{\textit{free}} N^{x}_{\textit{free}} + \alpha _{\textit{occ}} N^{x}_{\textit{occ}}, \end{aligned}$$

where $N_c^x$ is the number of cells observable from *x* of class *c*, and $\alpha _{\textit{unk}}> \alpha _{\textit{free}} > \alpha _{\textit{occ}} \ge 0$.

The $\alpha _c$ coefficients are normalised such that a gain value of 1 represents every ray cast seeing purely unknown space, and a value of 0 representing the robot being completely enclosed by occupied space. The specific values of the $\alpha$ parameters that produce the best performance will depend on the structure and connectivity of the environment: for example, whether it is made up of constrained corridors or wide-open spaces.

Note that our OctoMap-based environment representation already is a 3D representation that we project to 2D. The exploration algorithms we propose also generalise naturally to 3D, thus extending our framework to 3D is straightforward.

## Modelling and Problem formulation

### Underlying model

We consider a set of environmental processes which are unknown to the robot a priori. These processes represent environmental phenomena that vary across the environment *X*, such as radiation levels or underwater currents, for which the robot can draw noisy sensor measurements at its current location.

#### Definition 6

*(Unknown Processes)* The unknown processes at environment *X* are defined as $f: X \rightarrow O$ where $O= \mathbb {R}^m$ is the observation space of *f*, with *m* being the number of modelled unknown processes. The robot can observe noisy sensor measurement $\omega : X \rightarrow \mathbb {R}^m$ of the form:

7 $$\begin{aligned} \omega (x)=f(x) + \epsilon , \end{aligned}$$

where $\epsilon =[\epsilon _1, \ldots , \epsilon _m]$ is an *m*-dimensional vector of Gaussian observation noise, i.e. $\epsilon _j \sim \mathcal {N}(0, \sigma ^2_j)$ for all $j \in \{1, \ldots , m\}$.

For the remainder of the paper, we will consider $m=1$, i.e., we assume there is a single unknown process. However, the methods proposed can easily generalise to more than one unknown process, either using a multi-output GP (Osborne et al., 2008) or multiple single-output GPs. The former assumes non-independent process dynamics, where learning about one process could improve predictions of another.

For the purpose of planning and navigation, we represent the environment as a set of *d* waypoints ${{\,\textrm{V}\,}}=\{x_1, \ldots , x_{ d } \} \subset X$, and consider a stochastic model of navigation between waypoints under the influence of the unknown process.

#### Definition 7

A *partially known state MDP (PKSMDP)* for navigation is a tuple $\mathcal {M}^O=\langle S^O,\overline{s^O}, A^O,T^O, C^O\rangle$ where:

- $S^O = {{\,\textrm{V}\,}}\times O$, i.e., the state space is composed of a location in the environment ${{\,\textrm{v}\,}}$ and the corresponding value $f({{\,\textrm{v}\,}})$ of the unknown process;
- $\overline{s^O}=(\overline{{{\,\textrm{v}\,}}}, f(\overline{{{\,\textrm{v}\,}}}))$, where $\overline{{{\,\textrm{v}\,}}}$ is the robot’s initial position;
- $A^O = V \times V$ is a set of navigation actions such that $(v,v^{\prime }) \in A^O$ if the robot can navigate from *v* to $v^{\prime }$ without visiting any other waypoint on the way;
- $T^O: ({{\,\textrm{V}\,}}\times O) \times A^O \times {{\,\textrm{V}\,}}\rightarrow [0,1]$, where $T^O((v,o), (v,v^{\prime }), v^{\prime \prime })$ is the probability of the robot reaching waypoint $v^{\prime \prime }$ given that it attempted to reach waypoint $v^{\prime }$ from *v*, and the value of the unknown process at *v* is *o*;
- $C^O: ({{\,\textrm{V}\,}}\times O) \times A^O \rightarrow \mathbb {R}_{\ge 0}$ is the cost function, where $C^O((v,o), (v,v^{\prime }))$ is the cost of attempting to navigate from waypoint *v* to waypoint $v^{\prime }$ when the value of the unknown process at *v* is *o*.

Note that a PKSMDP is *not* a standard MDP as formalised in Definition 1. In particular, the transition function is defined such that the action outcomes are a distribution over only the set of waypoints. This is because the underlying state is uniquely defined by the value of the waypoint *v*, i.e., $s^O=(v, f(v))$. Furthermore, since *f* is unknown and the robot is only able to make noisy observations, the PKSMDP cannot be directly used for planning. Finally, note that $S^O$ is not a discrete set because its component *O* is continuous. In Sect. 5, we will propose a model that estimates the PKSMDP.

Previous approaches generally model the unknown process as a cost function rather than as part of the state. By including unknown process values as part of the state, the PKSMDP can model the impact of these process values on the robot dynamics.

We also consider a safety function defined over the values of the known and unknown state features.

#### Definition 8

A safety function is a mapping $\phi :{S}^O \rightarrow \{0,1\}$ where $\phi ({s}^o) =1$ when *s* is considered safe and 0 otherwise. The sets of safe and unsafe states can then be defined as $\textit{safe}_{\phi } = \{{s}^o \in {S}^O \mid \phi ({s}^o) = 1 \}$ and $\textit{unsafe}_{\phi } = {S}^O {\setminus } \textit{safe}_{\phi }$.

A simple safety function which depends only on a single unknown value state feature could be an upper-bound $b \in \mathbb {R}$ on the value of that feature, as used in Sect. 8.1.1. However, our approach allows for safety to be defined as an arbitrary Boolean function over the state-space of the PKSMDP, something which is not possible with existing approaches. We demonstrate such a safety function in Sect. 8.1.2.

### Problem formulation

We now pose the two problems addressed in this paper. First, we assume that the area has been previously spatially mapped and there is a known PKSMDP defined over the mapped area. The goal is to accurately estimate *f* whilst remaining safe.

#### Problem 2

Let $\mathcal {M}^O=\langle S^O,\overline{s^O}, A^O,T^O, C^O\rangle$ be a PKSMDP and $\phi :{S}^O \rightarrow \{0,1\}$ a safety function. Estimate the unknown process $f: X \rightarrow O$ whilst avoiding states ${s}^o = (v,o)$ where ${s}^o \in \textit{unsafe}_{\phi }$.

In Problem 2, we know the underlying spatial structure of the environment, and assume it has been discretised into a set of relevant waypoints. This discretisation can be achieved manually by a designer, or automatically, e.g. by employing Voronoi diagrams or gridding the space, removing edges and cells that intersect with the obstacles. Quantifying the quality of our estimate depends on the robot’s objective and the approach used for estimation, thus we deliberately leave it undefined in the problem definition above. In our proposed approach, we use the variance of the GP predictions of *f* on the waypoints, as will be explained in Sect. 6.2.

Second, we remove the assumption of a known map – and hence of a known PKSMDP – and consider the problem of safely exploring and mapping the environment. To do so, we assume the robot is equipped with a sensor (e.g. lidar) that is able to build an occupancy map incrementally, which is in turn used to classify voxels as unknown, occupied or free as described in Sect. 3.3.

#### Problem 3

Let *X* be a bounded environment, $\mathbb {M}$ a grid map representation of *X*, and $\phi :{S}^O \rightarrow \{0,1\}$ a safety function. Classify as much of $\mathbb {M}$ as possible as being in free or occupied, whilst avoiding states ${s}^o = (v,o)$ where ${s}^o \in \textit{unsafe}_{\phi }$.

To solve Problem 3, we will adapt the solution of Problem 2 to incrementally build and solve estimates of a PKSMDP rather than consider the exploration of a fixed underlying PKSMDP.

## GP-based PKSMDP estimation

Central to our approach is the construction of an MDP that estimates the unknown process represented in the PKSMDP given the data already observed by the robot. Our approach to do so is to use a GP, trained on observations up to timestep *t*, to iteratively estimate the mapping *o* between known state feature values and their corresponding unknown state feature values.

We start by partitioning the domain of the unknown process *O* into a finite set of intervals of possible values of the unknown process, allowing us to abstract the continuous aspect of the problem.

### Definition 9

We consider the finite partitions of *O* defined as the sets of intervals $\mathcal {I}$. Given $o \in O$, we write *I*[*o*] to denote the interval in $\mathcal {I}$ that contains *o*.

Partitioning the continuous space of the unknown process into a finite number of intervals enables the use standard solution techniques for MDPs, removing the need to consider continuous state spaces. As usual in this kind of discretisation, increasing the number of intervals leads to a more fine grained representation of the unknown process, at the cost of increased computational complexity.

We consider the GP posterior over these intervals.

### Definition 10

Let $\mathcal {D}$ be a dataset of observations of the unknown process *f*, $v_* \in {{\,\textrm{V}\,}}$ be a waypoint, and $I \in \mathcal {I}$ be an interval. The probability of the value of unknown process in $v_*$ being in interval *I* is defined as:

8 $$\begin{aligned} P^{\mathcal{G}\mathcal{P}}(f(v_*) \in I \mid \mathcal {D}) = \int _{o \in I} P^{\mathcal{G}\mathcal{P}}(f(v_*)=o \mid \mathcal {D})\,do. \end{aligned}$$

We can now define the *Estimated MDP* (EstMDP), which encodes both the probabilistic transition function of the PKSMDP and the GP model given the observed dataset $\mathcal {D}$.

### Definition 11

Let $\mathcal {M}^O=\langle S^O,\overline{s^O}, A^O,T^O, C^O\rangle$ be a PKSMDP, $\mathcal {I}$ be the set of intervals, and $\mathcal {D}$ be a dataset of observations of the unknown process *f*.

The estimated MDP is a tuple $\mathcal {M}^\mathcal {D}=\langle S^\mathcal {D},\overline{s^\mathcal {D}}, A^O,T^\mathcal {D}, C^O\rangle$, where $T^\mathcal {D}: ({{\,\textrm{V}\,}}\times \mathcal {I}) \times A^O \times ({{\,\textrm{V}\,}}\times \mathcal {I}) \rightarrow [0,1]$, where:

- $S^\mathcal {D}=V \times \mathcal {I}$, i.e., the state space are pairs of waypoints and intervals over the unknown process value at those waypoints;
- $\overline{s^\mathcal {D}}=(\overline{v}, I[\omega (\overline{v})])$, where $\overline{v} \in V$ is the initial waypoint of the robot;
- The transition function is defined as
  9 $$\begin{aligned} T^\mathcal {D}&((v,I), (v,v_g), (v^{\prime }, I^{\prime })) = \nonumber \\&T^O((v,I), (v,v_g), v^{\prime }) P^{\mathcal{G}\mathcal{P}}(f(v^{\prime }) \in I^{\prime } \mid \mathcal {D}), \end{aligned}$$
  where $T^O\left( (v,I), (v,v_g), v^{\prime }\right)$ is the average of $T^O((v,o), (v,v_g), v^{\prime })$ along interval $I=[l,u]$:
  10 $$\begin{aligned} T^O((v,I),&(v,v_g), v^{\prime })=\nonumber \\&\frac{\int _{l}^u T^O((v,o), (v,v_g), v^{\prime }) do}{u-l}. \end{aligned}$$

**Fig. 1:**
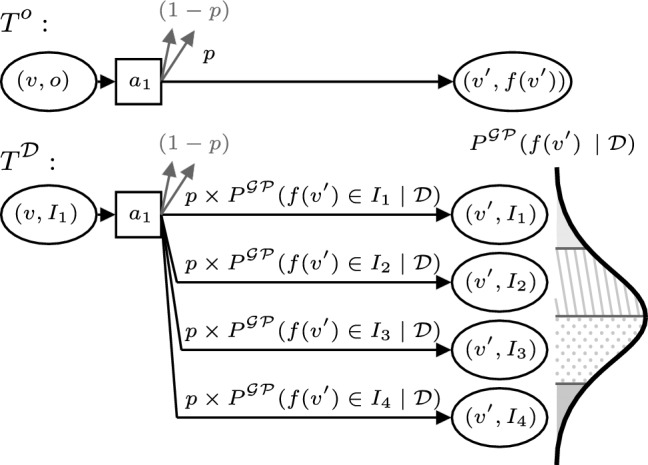
Construction of the estimated MDP transition function $T^\mathcal {D}$, combining the PKSMDP probabilistic transition function $T^O$ and GP predictive posterior given the dataset

The EstMDP defines a transition function for which we can plan for using standard techniques. It does so by (i) defining the transition function over intervals of *O* rather than *O* itself, by averaging it along each interval; and (ii) completing the transition definition, by weighting the transition probabilities in $T^O$ the GP estimate with the GP posterior over each possible interval, given the set of observed data $\mathcal {D}$. Figure 1 depicts an example $T^\mathcal {D}$.

The EstMDP is at the core of the exploration algorithms presented in the next two sections. To reason about safety on the EstMDP, we define the set of unsafe EstMDP states.

### Definition 12

Let $\mathcal {M}^\mathcal {D}$ be an EstMDP. The set of unsafe states of $\mathcal {M}^\mathcal {D}$ is defined as

11 $$\begin{aligned} S^{\textit{unsafe}}= \{(v,I) \in S^\mathcal {D} \mid \exists o \in I \text { s.t. } \phi ((v, o)) = 0 \}, \end{aligned}$$

and the set of safe states of $\mathcal {M}^\mathcal {D}$ is defined as

12 $$\begin{aligned} S^{\textit{safe}}= S^\mathcal {D} \setminus S^{\textit{unsafe}}. \end{aligned}$$

States (*s*, *I*) are considered unsafe if they are unsafe for any $o \in I$. This is an overestimate of the set of unsafe states, which in turns lead to a conservative definition of safe states. For convenience, we also define the set of safe intervals for a specific waypoint *v*:

13 $$\begin{aligned} \mathcal {I}^{\textit{safe}}(v) = \{I \in \mathcal {I} \mid (v,I) \in S^{\textit{safe}}\}. \end{aligned}$$

## SafeEstMDP-Process: modelling an unknown hazard

In this section, we present our approach to Problem 2, i.e., when we assume a known underlying navigation graph *V* and our goal is to predict *f* as well as possible. Given that we use a GP to estimate *f*, we stop exploration when the predictive variance of the GP is within a bound for all waypoints in *V* that can be reached safely.

### Algorithm description

**Fig. 2:**
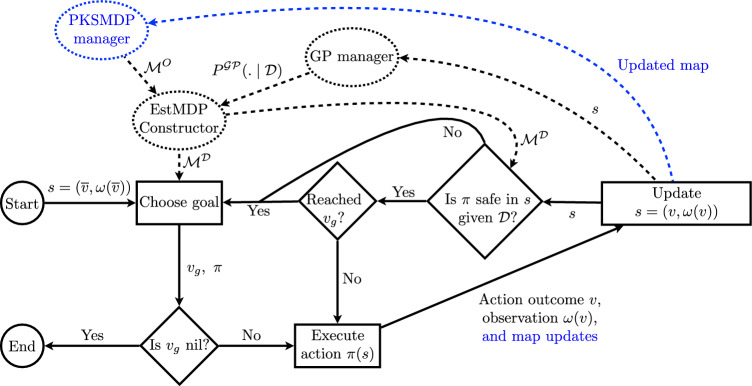
Flow diagram of the exploration method. Full lines represent main algorithm loop, and dashed lines represent structures responsible for data gathered during deployment and construction of the EstMDP given that data. Parts in blue are specific to the approach presented in Sect. 7 (Color figure online)

The flow diagram (Fig. 2) provides a high-level description of the exploration approach. At each exploration step, the robot uses its current knowledge of the PKSMDP to determine which *goal* state it should next visit in order to best improve its knowledge of other uncertain states. It uses the EstMDP to represent its current knowledge of the PKSMDP and makes decisions based on this estimation.

While executing the current policy $\pi$ that has been chosen to reach the current goal state, the robot carries out a *policy safety check* at each state. Carrying out the safety check allows the robot to replan when the probability of safely reaching the goal falls below the threshold $p_{\min }$. This check is particularly key when the transition function of the PKSMDP depends on the value of the unknown process, as the policy may entirely fail to reach the goal state if the values of the unknown process are not as the algorithm expected at goal selection time.

Constructing the EstMDP always uses the most up-to-date GP posterior, which is maintained within the GP manager module, and the PKSMDP given as input. In the next section, we will discuss how we can also build an PKSMDP incrementally, based on occupancy data sensed by the robot.

**Algorithm 1:**
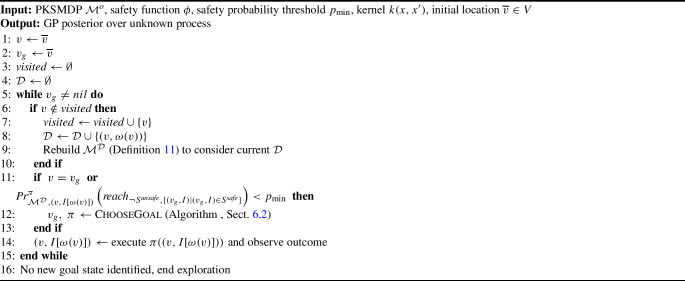
Safe exploration (SafeEstMDP-Process)

**Algorithm 2:**
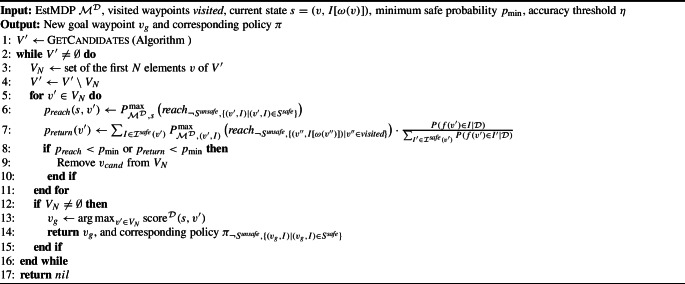
ChooseGoal

We provide further detail on the approach in Algorithm 1. The algorithm receives a PKSMDP $\mathcal {M}^o$ to be explored, a safety function $\phi$, a minimum safety probability $p_{\min }$, a GP kernel *k* and an initial location $\overline{v}$.

The exploration algorithm repeatedly finds new goal states to explore, until no more goals are available (line 5). The algorithm maintains a set of waypoints $\textit{visited}$ that have already been explored (and must therefore be safe) and a dataset $\mathcal {D}$ of observations of the unknown process at the explored waypoints. These are updated whenever the robot visits a new state (lines 7–8).

Before executing an action from the current policy, the robot checks whether the policy can continue being executed safely from the current state. It does this by evaluating the same constrained reachability problem that was used to select the current goal state, but now from the current state. Evaluating $\pi$ in the policy safety check is significantly less computationally demanding than generating $\pi$ as part of ChooseGoal. When the policy safety probability falls below a user-defined threshold $p_{\min }$ or the robot reaches its current exploration goal (line 11), then a new goal state must be selected. Where the PKSMDP transition and/or cost function are dependent on unknown value state features, one may also check the expected cumulative cost of continuing to execute $\pi$, and abandon the current goal if the cost grows significantly beyond the expected cost calculated at goal selection time.

The ChooseGoal routine and the policy safety check make use of the EstMDP $\mathcal {M}^\mathcal {D}$, obtained from $\mathcal {M}^o$ and the GP posterior given the dataset $\mathcal {D}$, as explained in Definition 11. The robot uses $\mathcal {M}^\mathcal {D}$ to choose a new waypoint to be explored and computes the corresponding policy, according to ChooseGoal (line 12, Algorithm 2 to be explained next). Finally, in line 14, the robot executes the action prescribed by its current policy, observes the outcome location, senses the value of the unknown process at that location, and updates the new state accordingly.

### Choice of goal waypoint

The choice of goal waypoint makes use of the EstMDP model, and is detailed by Algorithm 2. A good goal waypoint should provide information when observed (i.e. have a high predictive variance before observation) relative to the cost taken to reach it and, crucially, be safe to reach and return from.

**Algorithm 3:**
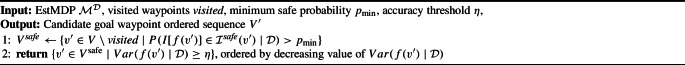
GetCandidates

In line 1 the set of candidate goal waypoints is generated by GetCandidates, as defined in Algorithm 3. This set consists of unvisited waypoints that (i) are considered safe with high probability and (ii) have a GP predictive variance greater than the accuracy threshold $\eta$. We then enter a loop where we analyse batches of *N* waypoints (lines 3 and 4).

Because Algorithm 3 orders candidates in $V^{\prime }$ by highest predictive variance, this will be the *N* waypoints in $V^{\prime }$ where the GP is less accurate. Each of these waypoints $v_g$ is checked for *reachability* and *returnability* probabilities. Specifically, the reachability probability (line 6) is defined as the maximum probability of safely reaching the set of states in $\mathcal {M}^\mathcal {D}$ corresponding to $v_g$ from the current state *s*. The returnability probability (line 7) is computed as the weighted average of the probabilities of safely returning from one of the states in $\mathcal {M}^\mathcal {D}$ corresponding to $v_g$ to a state corresponding to an already visited (hence, safe) waypoint. For the returnability check it is assumed that the initial state is safe, hence the normalisation at the end of line 7. If either of these probabilities is below the safety threshold $p_{\min }$, they are removed from the current batch of candidates (lines 8 and 9). Then, if there are still waypoints in the current batch of candidates, the remaining waypoint with the highest score (we propose a scoring function in the next section) is returned as the new goal waypoint (lines 12–14). The policy returned is the policy generated from the reachability check for the chosen goal waypoint. If the current batch of candidates is empty, the process is repeated for the next *N* highest variance waypoints. If no waypoint passing the reachability and returnability checks is found, then all waypoints that could be safely explored have been visited, and the algorithm returns *nil*, finishing the exploration (line 17).

**Goal scoring function.**  The goal scoring function $\text {score}^\mathcal {D}: S^\mathcal {D} \times V \rightarrow \mathbb {R}$ is designed to indicate how beneficial a waypoint *v* would be to visit and observe from current state *s*, given the current observed dataset $\mathcal {D}$. This score should take into account the GP’s predictive variance at *v*, the optimal expected cost to safely reach a state in $\{(v, I) \mid (v, I) \in S^{\textit{safe}}\}$ from current state *s*, and the reachability/returnability probabilities for *v*. We propose the following scoring function:

14 $$\begin{aligned} {\text {score}}^{\mathcal {D}}&(s, v) = \nonumber \\&Var(f(v) \mid \mathcal {D}) \times \nonumber \\&\textit{E}_{\mathcal {M}^\mathcal {D},s}^*\left( {\textit{cumul}_{\lnot {{S^{\textit{unsafe}}}},{\{(v, I) \mid {(v, I) \in S^{\textit{safe}}}\}}}}\right) ^{-\gamma _1} \times \nonumber \\&(p_{reach}(s,v)p_{return}(v) - p_{\min }^2)^{\gamma _2}, \end{aligned}$$

where the parameters $\gamma _1$ and $\gamma _2$ provide relative weightings on different parameters.

**Fig. 3:**
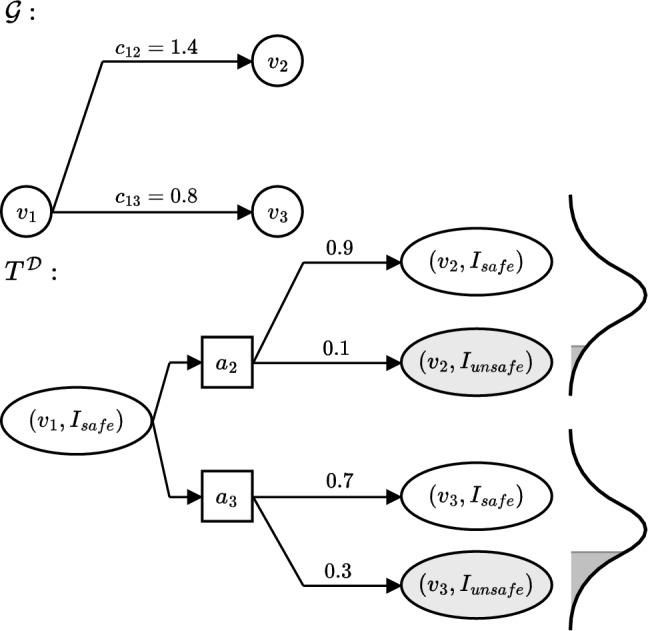
An example of a navigation graph $\mathcal {G}$ and corresponding Estimated MDP transition function $T^\mathcal {D}$, defined based on an interval set $\mathcal {I} = \{I_\text {safe}, I_\text {unsafe}\}$. Numerical values are derived from the GP posterior given $\mathcal {D}$. Navigation graph edges are annotated with costs, transition function edges with transition probabilities

Figure 3 shows an example EstMDP that might be used by SafeEstMDP-Process. In this scenario, starting at $v_1$ and supposing a safety threshold of $p_{\min }=0.5$, the selection of the next goal state (between either $v_2$ or $v_3$) would weigh up several factors: the cost to reach $v_2$ is higher, but taking action $v_3$ comes with a significantly higher risk of ending up in an unsafe state according to the GP. Given that the GP variances are also similar, $v_2$ would likely be selected as the next goal node, whereas $v_3$ would only be selected if its variance were significantly higher.

## SafeEstMDP-Map: exploring an unknown environment

In this section, we present our approach to Problem 3, i.e., when there is no assumption over the underlying map and the goal is to safely explore a bounded environment *X* represented by a grid map $\mathbb {M}$, and classify, as free or occupied, as many cells in $\mathbb {M}$ as possible. For this problem, we will incrementally build the navigation MDP $\mathcal {M}^O$ (Definition 7) and stop exploration when the volumetric gain associated to all safely reachable waypoints in *V* is less than a specified threshold.

### Algorithm description

There are two main differences with regards to the approach presented in the previous section: (i) there is a navigation graph construction process running in parallel with Algorithm 1. Instead of receiving a complete PKSMDP a priori, Algorithm 1 constructs an updated $\mathcal {M}^O$ whenever it needs to build an estimated MDP $\mathcal {M}^\mathcal {D}$, making use of the most up-to-date navigation graph representation of the currently explored environment; and (ii) the candidate waypoint selection, scoring function and stopping condition of ChooseGoal (Algorithm 2) are updated for the new exploration objective of safely mapping the environment. The blue part of the diagram in  Fig. 2 highlights this additional step.

### Incremental navigation graph construction

Our exploration system requires a mechanism for incrementally growing $\mathcal {M}^O$ based on local sensor measurements. This mechanism assumes that the robot navigation is *deterministic*, i.e., the transition function is such that $T^O((v,o), (v,v^{\prime }), v^{\prime \prime })= 1$ if $v^{\prime \prime }=v^{\prime }$, and 0 otherwise. Furthermore, we consider the MDP cost to be based on the Euclidean distance, i.e., $C((v,o), (v,v^{\prime }))=\Vert v,v^{\prime }\Vert _2$.

The deterministic navigation assumption is used so we do not need to estimate the outcome distribution of traversing a specific edge from sensor data, which is outside the scope of this paper.

However, if we have a mechanism that can do so, then our approach can deal with stochastic navigation, as there is nothing in the exploration algorithm that assumes deterministic dynamics. A PKSMDP with a deterministic transition function and Euclidean distance cost can be fully defined by its underlying graph $\mathcal {G}=(V, A^O)$. In the remainder of this subsection, we describe how to incrementally build $\mathcal {G}$. Note that any other cost function that maps pairs of waypoints to a positive number can be considered; we use Euclidean distance for ease of presentation. Furthermore, whilst we consider ray casting in a grid map which is derived from lidar scans, any other approach to calculate volumetric gain could be used. The graph-extending process is described in Algorithm 4.

**Algorithm 4:**
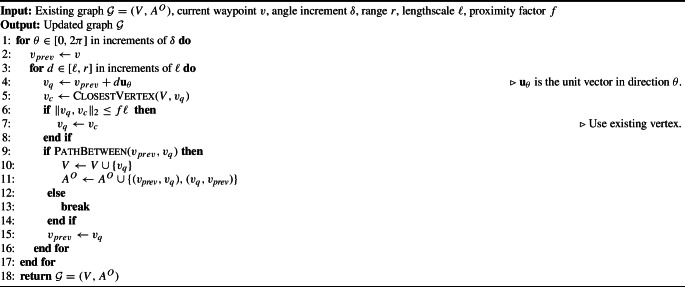
ExtendGraph

The algorithm casts rays outwards from the robot’s current location *v* in all directions within a horizontal plane, in angular increments of $\delta$. Along each ray, stepping outwards based on a lengthscale $\ell$, a new query waypoint $v_q$ is created (line 4). If $v_q$ is too close to the nearest pre-existing waypoint $v_c$, then the nearby waypoint is used as the query waypoint instead (line 7). Then, the occupancy map is queried to check if there is a path through free space between the previous query waypoint $v_{prev}$ and $v_q$ (line 9). If such a path exists, $v_q$ is added to the vertices in the navigation graph, and a bidirectional edge is added linking it to the previous vertex. Otherwise, the current chain of waypoints ends, as it is presumed blocked by an obstacle. In this way, the algorithm constructs chains of waypoints outwards from the current waypoint *v* through free space, connecting them to existing nearby waypoints whenever possible.

**Fig. 4:**
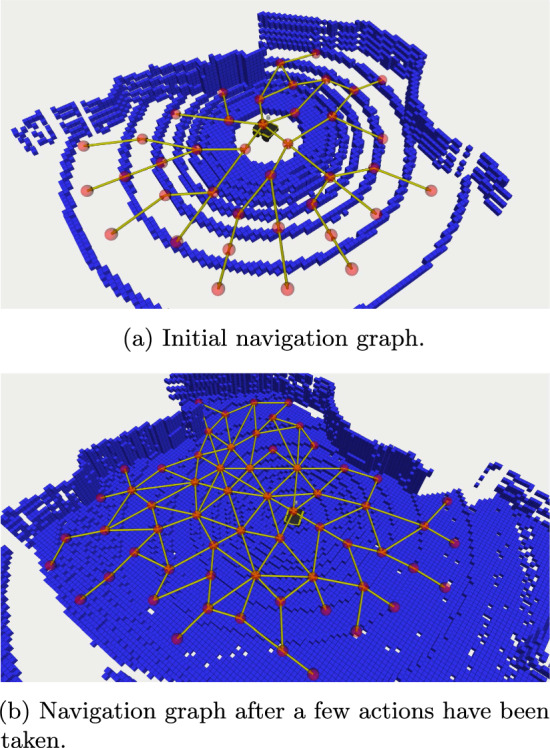
Navigation graph construction on a Clearpath Jackal robot simulated in Gazebo. Red spheres represent waypoints, yellow lines traversal actions (Color figure online)

Figure 4 demonstrates the navigation graph construction algorithm in action, showing the graph itself as well as a 3D occupancy map obtained from a Clearpath Jackal robot. The initial navigation graph (Fig. 4a) has edges radiating a few metres outwards from the robot’s initial location, in all directions except where the lidar’s line of sight is obstructed. Figure 4b shows a more developed navigation graph after a few actions have been taken.

### Choice of goal waypoint

**Algorithm 5:**
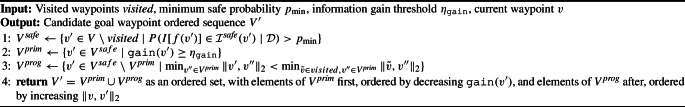
GetCandidates (SafeEstMDP-Map)

The candidate goal selection routine in ChooseGoal (Algorithm 2, line 1) now calls the SafeEstMDP-Map version of GetCandidates (Algorithm 5). The *primary* candidate set of states (line 2) is similar to the one returned by Algorithm 3. The only two differences are: (i) replacing the GP predictive variance term with volumetric gain to reflect the information gain objective, and (ii) specifying a minimum volumetric gain $\eta _{\texttt {gain}}$ that a waypoint must have to be considered as a primary candidate.

However, Algorithm 5 now also returns *progression candidates* (line 3), which are waypoints that progress the robot towards primary candidates. These are states for which $\texttt {gain}(v) < \eta _{\texttt {gain}}$, but visiting that state would decrease the minimum distance between the visited set of states and any primary candidate state. The addition of progression candidates is necessary because of the decoupling of information gain from the safety function. For most useful GP kernels, taking measurements increasingly closer to a primary candidate state will provide more information about that state’s safety.

**Fig. 5:**
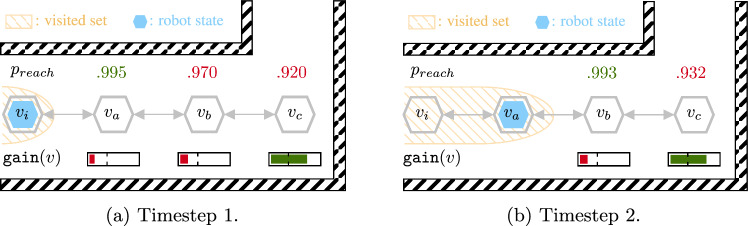
Robot progress towards high-information-gain states using progression candidates (Sect. 7.3)

Figure 5 shows an example of this behaviour. The robot is at the start of a row of waypoints along a corridor, ending in a $90^\circ$ corner. At the first timestep (Fig. 5a), the robot is at $v_i$. For this example, $p_{\min } = 0.99$. The waypoint $v_c$ provides a view around the corner, so has a high information gain and is a primary candidate. However, as it is several navigation edges away from the robot, $p_{reach}(v_i,v_c) < p_{\min }$: the robot will not choose $v_c$ as a goal state. The waypoint $v_a$ is safely reachable, but has information gain below the minimum threshold so cannot be a primary candidate. However, $v_a$ is a progression candidate because visiting it would decrease the minimum distance between the visited set of states and $v_c$. At timestep 2, the robot has chosen $v_a$ as its new goal and transitioned to it. Having taken a measurement at $v_a$, it is now more certain that $v_b$ and $v_c$ are safely reachable. It can then continue to choose $v_b$, which is now safely reachable with probability greater than $p_{\min }$, as a new goal state and progress further towards $v_c$. Without the addition of progression candidates, the robot would be unable to move along the corridor in this manner.

**Goal scoring function.**  Our scoring function is designed to encourage efficient exploration of the unknown space – a waypoint *v* is beneficial to observe if its volumetric gain is high, as this indicates it would likely be helpful in growing the robot’s map of the environment. Thus, we replace the GP variance component in Eq. 14 with the information gain term $\texttt {gain}(v)$ defined in Eq. 6. This mirrors Eq. 14, except with the exploration term now driven by increase of information over the map rather than minimisation of GP variance:

15 $$\begin{aligned} {\text {score}}^{\mathcal {D}}&(s, v) = \nonumber \\&\texttt {gain}(v) \times \nonumber \\&\textit{E}_{\mathcal {M}^\mathcal {D},s}^*({\textit{cumul}_{\lnot {{S^{\textit{unsafe}}}},{\{(v^{\prime }, I) \mid {(v^{\prime }, I) \in S^{\textit{safe}}\}}}}})^{-\gamma _1} \times \nonumber \\&(p_{reach}(s,v)p_{return}(v) - p_{\min }^2)^{\gamma _2}. \end{aligned}$$

Again, $\gamma _1$ and $\gamma _2$ specify the relative weightings of the components.

## Experiments

We present a series of experiments in simulation to demonstrate the performance of the SafeEstMDP-Process and SafeEstMDP-Map algorithms.

For all experiments, the MDP is solved via PRISM (Kwiatkowska et al., 2011) using nested value iteration (Lacerda et al., 2019), and the GP framework used is GPy (GPy, 2012).

### Evaluation domains

We use two sets of domains for evaluation of our algorithms in simulation. In the first set of domains (Sect. 8.1.1), the unknown process is nuclear radiation, and the transition dynamics are independent of the unknown process. The second set (Sect. 8.1.2) considers exploration in the presence of unknown ocean currents, which affect the transition dynamics.

#### Nuclear

We consider the safety hazard of gamma radiation exposure, which can be harmful at high levels to both robots and humans. Furthermore, standard gamma radiation survey sensors only provide noisy, local measurements. This matches our locally observable safety formulation.

**Fig. 6:**
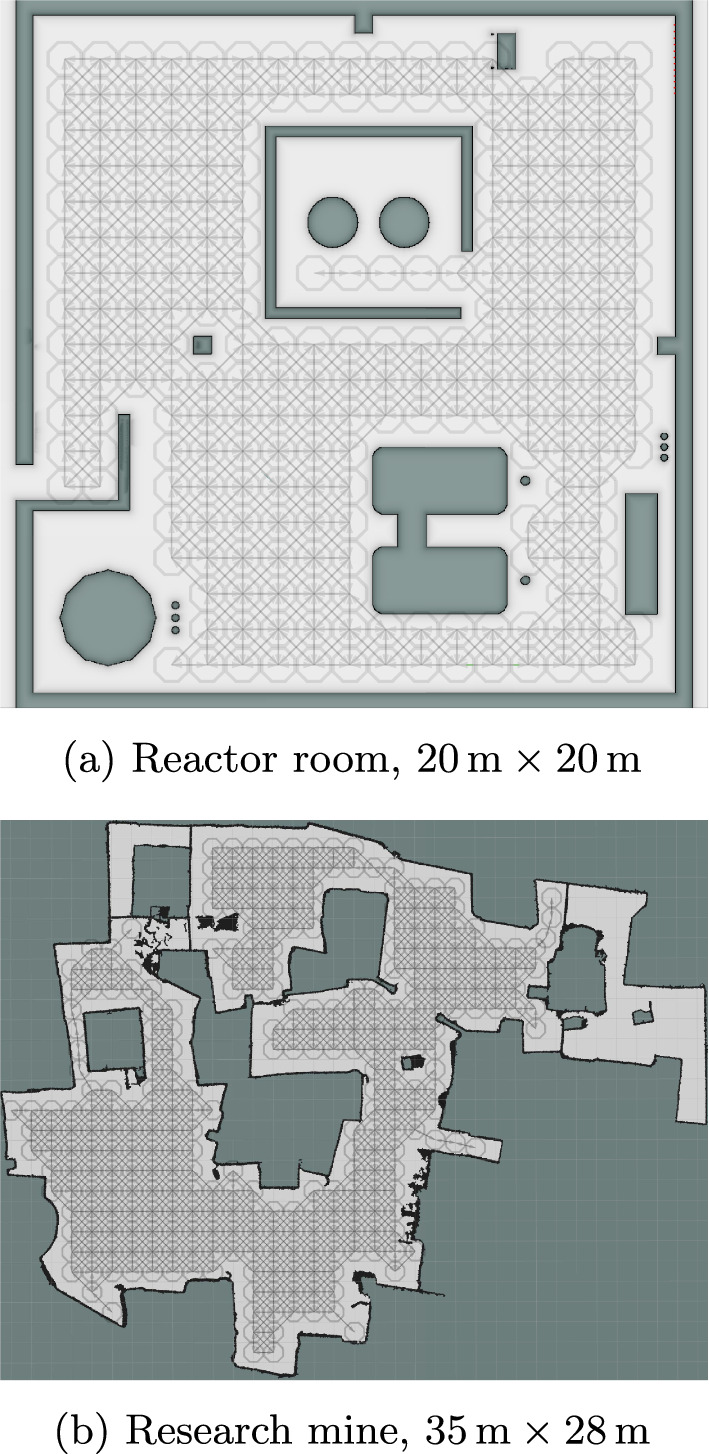
2D occupancy maps of both simulated experiment domains, with the navigation map used to build the PKSMDP for SafeEstMDP-Process shown in grey (Color figure online)

We evaluate with the nuclear radiation hazard in two simulated domains, illustrated in Fig. 6.

1. *Reactor room*, Fig. 6a: a simulated world representing a 20 m $\times$ 20 m nuclear reactor room, from Wright et al. (2021).
2. *Research mine*, Fig. 6b: A 35 m $\times$ 28 m map built from 3D SLAM in a real underground mine section at Corsham, UK.

The evaluation of SafeEstMDP-Process in Sect. 8.2 requires a PKSMDP to be provided. For each domain, PKSMDPs are pre-generated using 8-connected grid maps with $1\,\hbox {m}$ grid units, which can be seen in Fig. 6. In contrast, in Sect. 8.4 the PKSMDP is sequentially built over the map by SafeEstMDP-Map.

**Simulation of radioactive environments.** 

Radiation is simulated using $$1/r^2 $$ “solid angle” radiation physics (Wright et al., 2021). Radiation sources $$\{(\chi ^{src}_i, x^{src}_i)\}_{i=0}^{n_{src}}$$ have strength $$\chi ^{src}_i$$ and pose $$x^{src}_i$$. Source strength is the exposure value at a distance of 1 m. The radiation exposure $$\lambda (x)$$ at robot pose $$x \in X$$ from these radiation sources is then:

16 $$\begin{aligned} \lambda (x) = \sum _{i=1}^{n_{src}} \frac{\chi ^{src}_i}{\Vert x - x^{src}_i \Vert _2^2}. \end{aligned}$$

**Fig. 7:**
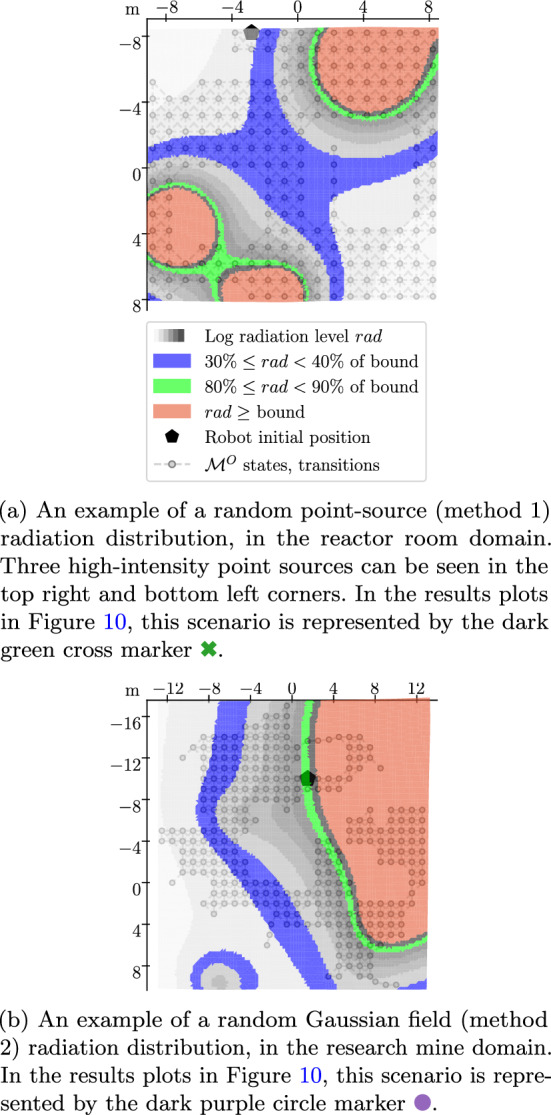
Two valid random radiation distributions, generated according to Sect. 8.1.1. A table of all radiation distributions used in Sect. 8.2 can be found in Appendix A.4

For each exploration scenario, a PKSMDP (Sect. 8.2) or a 3D simulated environment (Sect. 8.4) is combined with a randomly generated distribution of radiation sources. Sampled radiation distributions are discarded when they result in trivial exploration results with too much of the environment being safe or unsafe. In this work we generate radiation distributions that result in a minimum of 40% of the environment’s states being safely reachable from the initial state, and a maximum of 90%.

Random radiation fields are generated in both of the following ways, with each method contributing 50% of the evaluation environments:

1. Random point-source distribution: insert a randomly chosen number of sources ($5 \le n_{src} \le 30$), with uniformly random sampled poses across the environment’s domain, with randomly sampled *z* position values $z \in \{ 1.0, 1.5, 2.5 \}$ and randomly sampled strengths ${\chi ^{src}} \in \{ 250, 500, 1000, 2000, 5000 \}$. *x* and *y* position values are sampled uniformly within the bounds of the map $\pm {2}\,\hbox {m}$.
  An example of this type of radiation distribution can be seen in Fig. 7a.
2. Randomly generated Gaussian random field distribution: evenly cover the map with radiation sources at $z=1.0$ and draw their log strengths from a Gaussian random field.
  The Gaussian random field is generated with a radial basis function kernel, using uniformly sampled lengthscale hyperparameter $l \in \{ 3.0, 5.0, 7.0 \}$ and variance hyperparameter $\sigma \in \{ 20, 30, 50, 75 \}$.
  An example of this type of radiation distribution can be seen in Fig. 7b.

**GP modelling of radioactive environments.** 

To explore while ensuring safety with high probability, an exploration algorithm’s GP model must be able to well-model the ground-truth hazard function. Previous works (Silveira et al., 2018; West et al., 2021; Khuwaileh & Metwally, 2020) have demonstrated GP regression as capable of accurately modelling radiation fields in the real world. Silveira et al. (2018), West et al. (2021) log transforms the radiation level measurement data, in a similar manner to the log-warped GP model we describe below. Several features of radiation fields present challenges for standard GP regression. Firstly, the values of the radiation intensity function over 3D space may vary over several orders of magnitude. This is particularly evident when high-intensity radiation sources are present. Furthermore, since standard GP predictive Gaussian posterior distributions are unbounded, the GP will assign some probability to a radiation level $< 0$. This is clearly nonphysical, as the ground-truth radiation level is non-negative.

Secondly, the standard GP assumption of zero-mean Gaussian observation noise with input-independent standard deviation does not hold. At an abstract level, radiation measurements are based on counts of discrete detection events: for example, counts produced by a Geiger-Muller tube. Statistically these are samples from a Poisson distribution with rate parameter $\lambda$ being the ground-truth radiation level. Samples from this distribution have standard deviation $\sqrt{\lambda }$. Therefore, as the radiation level increases, the absolute measurement noise increases and the relative measurement noise decreases. Due to this effect and the non-linear behaviour of the Geiger-Muller tube, real-world radiation sensors are generally specified with a percentage rather than absolute reading accuracy (Knoll, 2010).

GPs with a non-Gaussian or heteroscedastic observation function are non-conjugate and therefore cannot be solved in closed form. Computationally intensive methods, including Markov Chain Monte Carlo sampling and Laplace approximation, are required (Williams & Barber, 1998).

To alleviate these issues, we follow the approach of Snelson et al. (2003) and use a log-warped Gaussian process regression. In this formulation, we model the log of the radiation level value $f\left( \log (rad)\right)$ with a GP. This transforms a normally distributed percentage observation noise with standard deviation $\sigma _\%$ in the radiation level into a normally distributed observation noise with standard deviation $\sigma$, where:

17 $$\begin{aligned} \exp \left( \log (rad) + \sigma \right)&= rad \cdot \left( 1 + \sigma _\%\right) ,\nonumber \\ \sigma&= \log \left( 1 + \sigma _\% \right) . \end{aligned}$$

The relative measurement noise formulation therefore better matches our sensor’s percentage measurement noise. Furthermore, the log-warped GP is also significantly better able to handle order-of-magnitude variation in the radiation level compared to a standard GP regression.

**EstMDP modelling of radiation levels.** 

In this domain, the unknown process $f: V \rightarrow \mathbb {R}$ is the value of the radiation level at each state. As the radiation level does not affect waypoint transitions, we need only define two intervals $\mathcal {I} = \{I_\text {safe}, I_\text {unsafe} \}$ where $I_\text {safe} = [0, \log (\lambda ^{\max })]$ and $I_\text {unsafe} = [\log (\lambda ^{\max }), \infty )$ and $\lambda ^{\max }$ is the radiation level safety bound. If a non-log GP were used, the EstMDP structure would be identical, with equivalent intervals $I_\text {safe} = [0, \lambda ^{\max }]$ and $I_\text {unsafe} = [\lambda ^{\max }, \infty )$. The exploration GP models the 1D radiation level at each state: $P^{\mathcal{G}\mathcal{P}}: \mathbb {R}^2 \rightarrow \text {Dist}(\mathbb {R})$. The GP input is $\{x, y\}$, in units of metres in a local coordinate frame.

#### Unknown currents

In this set of domains, the autonomous agent is an underwater autonomous vehicle (AUV) exploring the open ocean. The unknown process is the underwater current, which probabilistically affects the AUV’s transition dynamics. This is different from the setting of Sect. 8.1.1, where the PKSMDP transition function $T^O$ is deterministic and independent of *O*.

**Fig. 8:**
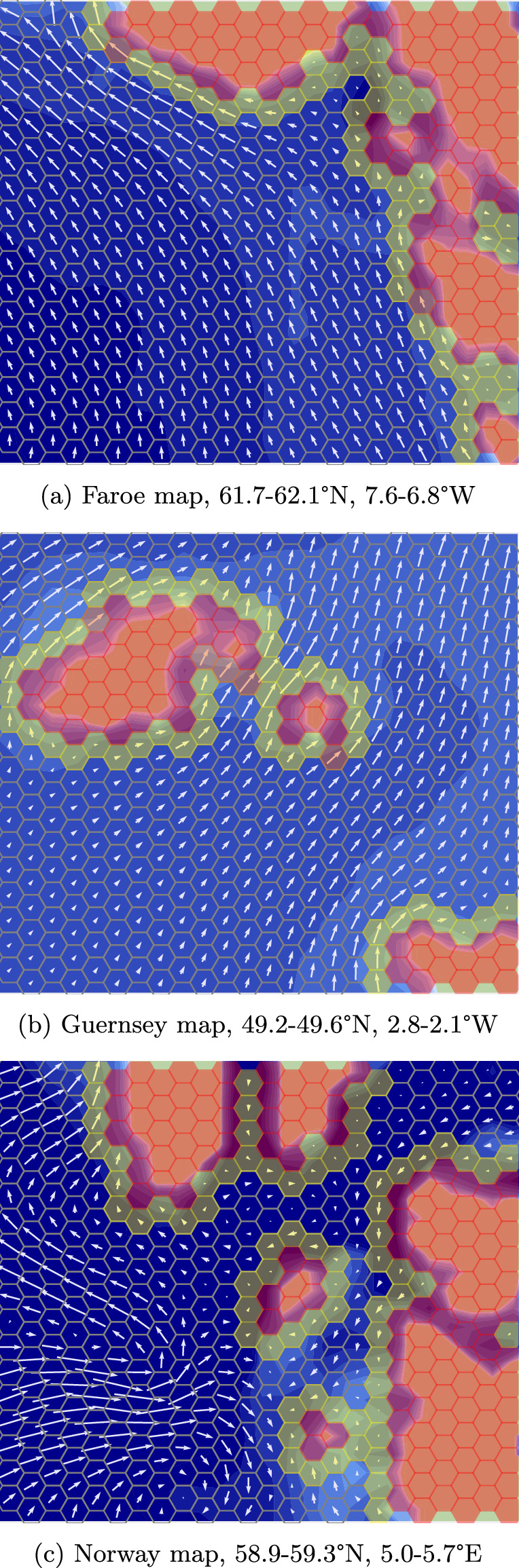
AUV domain areas, showing ground-truth currents (arrows) and state safety (hex cell colour) (Color figure online)

The AUV travels in a given direction by diving to a fixed depth and then travelling at a fixed speed relative to the water surrounding it, surfacing after a fixed length of time. Depending on the direction and magnitude of the water currents, this can result in the AUV surfacing in different locations. We discretise the ocean into a grid of hexagonal cells, as shown in Fig. 8. The figure illustrates the three evaluation domains, *Faroe*, *Guernsey*, and *Norway*, each of which is a 43.2 km $\times$ 37.4 km area covered by a $24 \times 18$ hex grid of side length ${1.2}\,\hbox {km}$.

The AUV fails its safety specification when it enters a cell with water depth $\le$ ${10}\,\hbox {m}$, as it is at risk of colliding with the seabed. These “*definitely unsafe*” states are known to be unsafe a priori, based on bathymetry data described below, and are shown in red in Fig. 8. The AUV also fails its safety specification when it enters a cell where the water current magnitude is too high in the direction of an a priori unsafe state. This would result in the AUV being carried into an unsafe state before it can dive again. States which may be unsafe depending on the current value at that state, “*maybe unsafe*” states, are shown in yellow in Fig. 8. Dark orange cells show “maybe unsafe” states which are in fact unsafe given the ground-truth current value at that state. This is an example of a more complex safety specification, which is defined on both known and unknown state feature values.

**Fig. 9:**
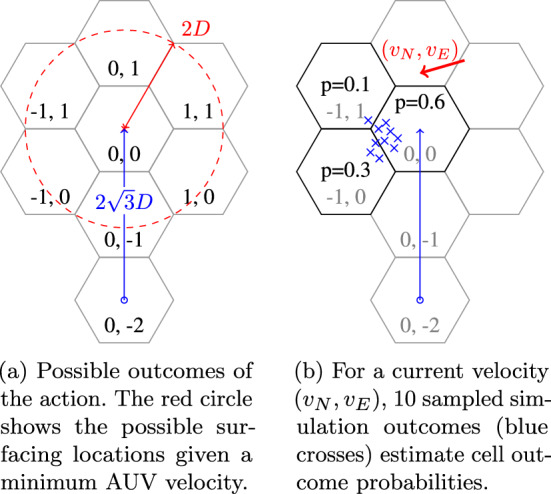
Illustration of possible and sampled outcomes of the AUV navigating from (0, −2) to (0, 0) in the hex grid

Actions are to attempt to travel 2 cells in one of the 6 hexagonal directions, with the AUV surfacing at the end of the action. An action in the north direction from cell $(0, -2)$ to cell (0, 0) is illustrated by the blue arrow in Fig. 9. To limit the number of possible action outcomes, we wish to ensure that the AUV can only surface in the target cell or one of its immediate neighbours. We do this by calculating the minimum required AUV velocity for the maximum current magnitude in the dataset.

As illustrated in Fig. 9a, the distance from an initial cell to an action’s target cell is $2 \sqrt{3} D$ where *D* is the hex cell side length. The time taken for the AUV to travel this distance is $2 \sqrt{3} D / v_\text {AUV}$ seconds. The maximum distance that the AUV can be carried by the current in this time is $v_{\text {max}} \times 2 \sqrt{3} D / v_\text {AUV}$, where $v_{\text {max}}$ is the maximum current velocity. If this distance is less than 2*D*, then the AUV can only surface in the target cell or one of its immediate neighbours. The minimum AUV velocity to ensure this is therefore $v_\text {AUV} > \frac{1}{\sqrt{3} v_{\text {max}}}$.

We assume that throughout the dataset the maximum current velocity in each axis is ${0.65}\,\hbox {m}\,\hbox {s}^{-1}$. We select $v_\text {AUV} =$ ${1.8}\,\hbox {m}\,\hbox {s}^{-1}$, as $1.8 > \frac{1}{\sqrt{3} \sqrt{2 \left( 0.65^2\right) }}$.

Action costs are the constant time taken to execute an action: $C((v,o), (v,v^{\prime }))=1$.

**Simulation of unknown currents and underwater autonomous vehicle.** 

Ground-truth bathymetry and water current velocity data is taken from the NORTHWEST_ SHELF_ANALYSIS_ FORECAST_PHY_004_013 dataset^1^ , which covers the northwest European shelf area. A kinematic AUV simulator (Budd et al., 2022) is used to forward simulate vehicle control, actuation and stochastic disturbances in a Monte Carlo manner, with a 1 Hz simulation frequency. The simulated AUV is a small, moving-mass propellor-driven vehicle with neutral buoyancy and noisy actuation. Water currents acting on the simulated vehicle are linearly interpolated from the ground-truth dataset points. The “true” outcome of a navigation action is sampled by running this simulator with ground-truth current values, as illustrated in Fig. 9b. This results in an implicit “ground-truth” MDP with probabilistic action outcomes.

**EstMDP modelling of unknown current navigation.** 

In this domain, the unknown process $f: V \rightarrow \mathbb {R}^2$ is the 2D vector of water current velocities at each state. Intervals $\mathcal {I}$ therefore represent a range of values for the north and east current velocity components $v_N$ and $v_E$. We define the intervals as the cartesian product of two 1D intervals, one for each component of the 2D vector, i.e. $\mathcal {I} = \mathcal {I}_N \times \mathcal {I}_E$ where $\mathcal {I}_N = \mathcal {I}_E$ for simplicity. We assume that the AUV noisily measures current velocity at states it passes through, using an onboard sensor or by analysing its end position after surfacing. The exploration GP is a coregionalised GP which models the 2D vector $(v_E, v_N)$ of east/north current velocity at each state: $P^{\mathcal{G}\mathcal{P}}: \mathbb {R}^2 \rightarrow \text {Dist}(\mathbb {R}^2)$. The GP input is $\{x, y\}$, in units of metres in a local coordinate frame.

To apply SafeEstMDP-Process to the AUV domain, we must define the interval-dependent transition function $T^O((v,I), (v,v_g), v^{\prime })$. We do not have a closed-form expression for $T^O((v,o), (v,v_g), v^{\prime })$ to use with Eq. 10: we can only sample navigation outcomes from the AUV simulator. We therefore build a *transition kernel* which estimates the probability of each possible outcome, relative to the cell the action was taken in, given the 2D interval over the current velocity at that start state. For a specific interval *I*, an evenly spaced grid of $10 \times 10$ current velocity values $(v_N, v_E)$ are taken from the interval. We run 20 repeats of the AUV simulator for each of these values, and record the resulting surfacing cell. Figure 9b shows 10 sampled outcomes for a specific $(v_N, v_E)$ value. This process gives us an estimate of the probability of each possible transition outcome given the interval *I*, and is independent of start state cell.

### SafeEstMDP-Process: mapping an unknown hazard

**Fig. 10:**
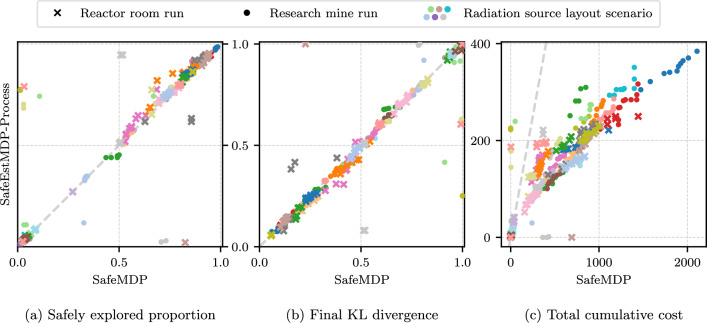
Scatter plots comparing SafeEstMDP-Process, on the y-axis, to SafeMDP, on the x-axis. The two algorithms are compared on **a** the proportion of the ground-truth safely reachable set the algorithm is able to correctly mark as safe, **b** the final KL divergence from the algorithm’s GP model to a “ground truth” GP model, and **c** the total cost the algorithm has incurred by the end of exploration. Total 400 runs of SafeEstMDP-Process and SafeMDP (2 domains, 20 randomly generated radiation layouts, 10 repeats). All runs terminated without breaking the safety specification. See Fig. 21 (appendix) for plots of all scenarios shown here

In this section, we compare SafeEstMDP-Process to SafeMDP (Turchetta et al., 2016), a prior MDP safe exploration algorithm which aims to determine the ground-truth maximum reachable safe set of states. To generate a navigation MDP and unknown process to explore, we generate 8-connected grid maps with side length ${1}\,\hbox {m}$ in the reactor room and research mine domains, combined with randomly generated radiation distributions according to Sect. 8.1.1.

For all experiments $p_{\min } = 0.99$, the goal selection weights are $\gamma _1 = 1.0$ and $\gamma _2 = 0.8$, the batch evaluation size $N = 8$ and $\eta = 0.01$. Unless otherwise stated, the GP kernel was an RBF with variance 1.0 and lengthscale ${2.0}\,{m}$. We also ran these experiments with a Matern 3/2 kernel, producing qualitatively similar results. The GP observation noise $\sigma ^2 = 0.0009$, corresponding to $\sigma _\% \approx 3\%$ (Eq. 17). The robot’s initial position is randomly sampled from a set of 4 waypoints. The safety bound $\lambda ^{\max } = 1000$.

Figure 10 compares SafeEstMDP-Process and SafeMDP across 400 runs for each algorithm: for each of the 2 domains, 20 radiation layouts are randomly generated and used for 10 repeats. Figure 10a shows the proportion of the ground truth safely reachable state space that each algorithm has correctly determined as safe, at the point of termination (larger is better). Similarly, Fig. 10b shows the KL divergence at termination between each algorithm’s GP model and a *ground-truth GP* trained on noiseless observations of every state in the PKSMDP (smaller is better). KL divergence is evaluated at all states in the PKSMDP, and is normalised relative to the KL divergence of the algorithm’s initial GP at the start of execution. Figure 10c shows the cumulative cost incurred by the robot at termination time. Each figure includes a grey, dashed equal performance line. Note the different axis scaling in Fig. 10c, which we use to make visualisation easier. The need to do so highlights how SafeEstMDP-Process clearly outperforms SafeMDP in terms of cost. Finally, for a given marker on these scatter plots, an illustration of the corresponding exploration scenario can be found in Appendix A.4.

The symmetry and strong diagonal distributions of 10a and 10b illustrate that SafeEstMDP-Process and SafeMDP are equally capable of exploring the safely reachable state space and building an accurate model of the unknown process. The major difference is seen in 10c: SafeEstMDP-Process is capable of achieving the same results with far lower cost incurred. This is particularly true when exploring MDPs with much of the state space safely reachable, e.g. the dark blue circle markers  at the top right of a and c, bottom left of b. In this scenario SafeEstMDP-Process incurs approximately $5 \times$ less cost than SafeMDP (note that the axes are not equally scaled).

**Fig. 11:**
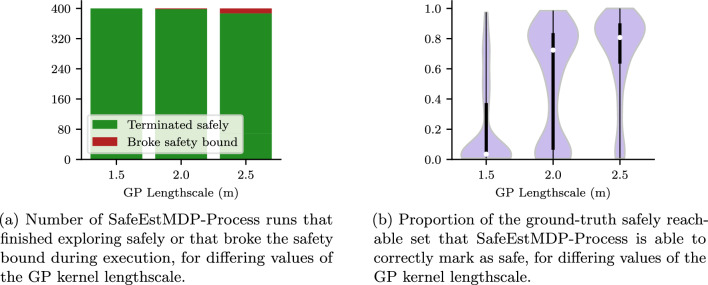
The effects of varying GP kernel lengthscale (x axis) on the behaviour of SafeEstMDP-Process. Total 400 runs per GP lengthscale value (2 domains, 20 randomly generated radiation layouts, 10 repeats)

The distribution of markers in these figures illustrates that, for both algorithms, it is harder to fully explore the safely reachable state space in some scenarios than in others. The dark blue circle marker  scenario consistently terminates with close to 100% of the safe reachable state space explored and close to 0 final KL divergence. Conversely, the dark purple circle marker  scenario is consistently barely explored, with the explored proportion close to 0%. As can be seen in the plot of this scenario in Appendix A.4 Fig. 21b, the initial location for the robot immediately neighbours an unsafe area. This results in the robot being unable to choose a location to move to while ensuring safety with sufficient certainty.

One scenario which shows more complex behaviour is that represented by the dark green cross marker . In this scenario, most runs terminate with close to 80% of the safe reachable area explored, but some terminate with less than 10%. This scenario is plotted in Fig. 7a, which shows that the robot initial location is at the start of a corridor leading to the rest of the map. To reach the rest of the map, the robot must travel towards a more dangerous area before rounding the corner. In some runs, the robot will sample higher-than-average noisy radiation level measurements. It may conclude that it cannot safely round the corner, and will terminate without exploring the rest of the map.

Overall, these results demonstrate SafeEstMDP-Process’s improved cost efficiency over SafeMDP. SafeEstMDP-Process is able to choose goal states that are multiple steps away from the currently explored set, while SafeMDP will only choose goal states neighbouring the currently explored set. By evaluating the safety and cost of paths to goal states, SafeEstMDP-Process is able to trade off exploration information gain vs reachability cost, so is able to explore the state space more efficiently. Considering multi-step paths to goal states also offers the option of taking measurements only at goal points (rather than every visited state), which is useful if measurements are expensive or time-consuming.

In Fig. 11 we analyse the effect of the GP kernel lengthscale hyperparameter on safe exploration with SafeEstMDP-Process. Figure 11a illustrates the number of runs that terminate safely compared to those that terminate by breaking the safety bound. Figure 11b shows distributions of the explored ground-truth safely reachable state space over many runs, this time depicted as a violin plot to facilitate comparison across the three tested lengthscale values.

It can be seen that a longer lengthscale value allows the exploration algorithm to reliably explore more of the ground-truth safely reachable state space, resulting in a higher concentration of runs with safely explored proportion close to 1.0 in 11b. With a lengthscale value of ${1.5}\,\hbox {m}$, almost all runs are unable to explore more than 20% of the safe state space.

However, the safety guarantees provided by GP safe exploration algorithms are entirely dependent on accurate modelling of the hazard function with the GP. Despite $p_{\min }$’s value of 0.99, a lengthscale of ${2.5}\,\hbox {m}$ does not model the radiation hazard function well enough, which yields overly aggressive robot behaviour. This results in the robot breaking the safety bound in 3.25% of the runs, compared to only 0.5% of runs for lengthscale ${2.0}\,\hbox {m}$.

**Fig. 12:**
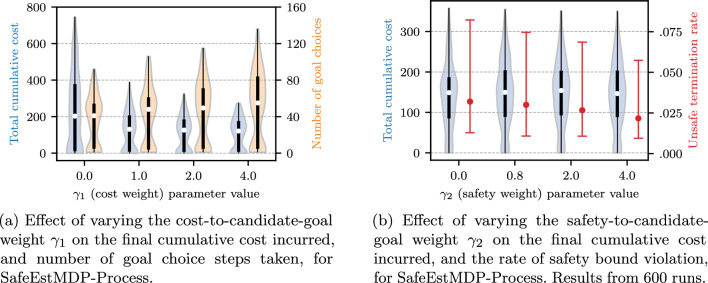
Ablation behaviour for SafeEstMDP-Process goal choice hyperparameters. Within violin plots, white dots show median values and thick black lines show interquartile ranges (Color figure online)

In Appendix A.3, we show the results from repeating these experiments using a GP without the log-warping, which results in a significantly higher rate of safety bound violation, since the model less accurately captures the true behaviour of the radiation hazard. Note that, because $p_{\min }$ attempts to bound the safety of an *individual* goal choice in SafeEstMDP-Process, $p_{\min }$ is not directly comparable with success rates over exploration episodes with many goal choice steps.

We carry out analysis of the effect of the $\gamma _1$ and $\gamma _2$ goal choice hyperparameters (Eq. 14) on SafeEstMDP-Process in Fig. 12. Figure 12a shows that as more priority is placed on finding low-cost goal states by increasing $\gamma _1$, the final cumulative cost incurred by the robot decreases. This is expected, as the robot is more likely to choose goal states that are closer to the current state, and likely takes a more efficient path throughout execution. As well as decreasing the relative importance of the other goal candidate score components, increasing $\gamma _1$ also increases the number of goal choice steps taken throughout exploration, as the robot is more likely to choose goal states that are closer to the current state. This will increase the computational cost of the algorithm.

Figure 12b shows a possible trend in safety violations as $\gamma _2$ increases. Confidence intervals are large as the proportion of safety violations is low, but the trend is consistent across the three tested values. Confidence intervals are produced by Jeffreys credible interval analysis using 600 runs across all radiation domain environments. The figure shows that the final cumulative cost incurred by the robot is largely insensitive to the value of $\gamma _2$. There was also no significant effect on the number of calls to the goal choice algorithm. This ablation used a ${2.5}\,\hbox {m}$ lengthscale value to ensure some safety violations occurred.

### SafeEstMDP-Process: exploration in the presence of unknown transition dynamics

In this section we demonstrate the ability of SafeEstMDP-Process to explore safely in the presence of unknown transition dynamics. Existing MDP safe exploration algorithms such as SafeMDP are not capable of reasoning about probabilistic and uncertain transition dynamics, so we have no baselines to compare SafeEstMDP-Process to.

Current velocity measurements have standard deviation ${0.03}\,\hbox {m}\hbox {s}^{-1}$ in each axis. The GP kernel is an RBF with variance 0.2 and lengthscale ${4}\,\hbox {km}$, which were found to be suitable for the NWES dataset area. The GP likelihood noise was fixed to $\sigma ^2 = 0.0009$. We define current value intervals $\mathcal {I}_N = \mathcal {I}_E = \{(-0.65, -0.25), (-0.25, 0.25), (0.25, 0.65)\}$, resulting in 9 intervals. For all AUV experiments $p_{\min } = 0.95$, the goal selection weights are $\gamma _1 = 1.0$ and $\gamma _2 = 0.8$, the batch evaluation size $N = 3$ and $\eta = 0.01$. “Maybe unsafe” states are unsafe if $v_E > 0.25$ and the state to the east is “definitely unsafe”, and similarly for $v_E < 0.25$ and a “definitely unsafe” state to the west. “Maybe unsafe” states are also unsafe if $v_E > 0.25$ and $v_N > 0.25$ and the state to the north-east is “definitely unsafe”, and similarly for the other 3 diagonal directions in the hex grid.

**Fig. 13:**
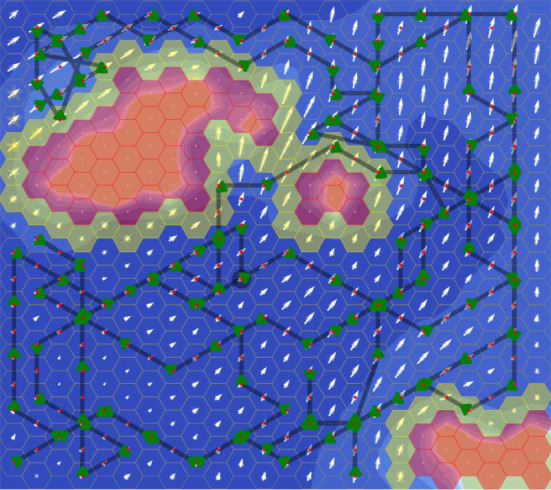
An illustrative execution history of SafeEstMDP-Process in the AUV domain. White arrows show the final GP model. Dive locations are marked by green arrows. See Fig. 8b for the ground-truth current field and safe set (Color figure online)

**Fig. 14:**
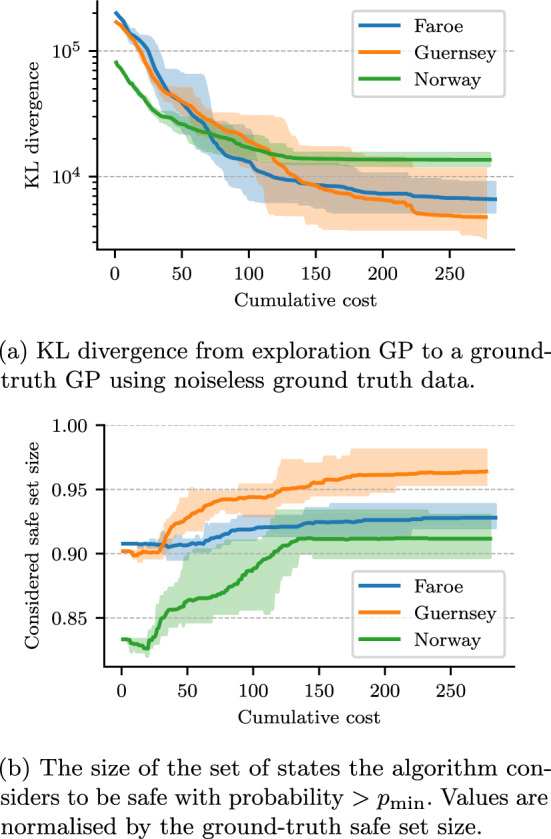
Exploration progress vs cumulative cost (time taken). For each map, the solid line shows mean value and shaded area shows the range of values across 10 repeats (Color figure online)

SafeEstMDP-Process was evaluated with 10 runs in each of the Faroe, Guernsey, and Norway maps. SafeEstMDP-Process did not visit an unsafe state in any of the 30 runs. Figure 13 shows an illustrative execution history of SafeEstMDP-Process in the Guernsey map, and Fig. 14 shows quantitative results.

Figure 14a illustrates the evolution in GP fit quality as the AUV incurs cost while exploring. GP fit quality is measured by Kullback–Leibler (KL) divergence from the exploration GP to a *ground-truth* GP trained on noiseless observations of the ground-truth current value at every hex cell. As the AUV explores and incurs more cost, the GP model becomes more accurate and the KL divergence therefore decreases.

Similarly, Fig. 14b shows the algorithm’s progress in identifying the ground-truth safe set of states. The algorithm starts off immediately considering a large proportion of the state space to be safe. This is because a majority of states ($\sim 83\%$ of states for Norway and $\sim 90\%$ for Faroe and Guernsey) do not neighbour an unsafe state so are therefore always safe. As the AUV explores, it becomes more confident in the (un)safety of “maybe unsafe” states, and the proportion of the state space considered safe increases.

SafeEstMDP-Process is able to identify most of the ground-truth safe set of states and build an accurate model of the unknown process in all three maps. The hardest map to explore is Norway, where the best runs were able to identify $\sim 93\%$ of the ground-truth safe set. This is because no runs successfully reached the top right corner of the map, which requires passing through a narrow channel with unknown state safety either side.

### SafeEstMDP-Map: exploring unknown environments

We evaluate the SafeEstMDP-Map algorithm in the two nuclear domains shown in Fig. 6, this time in a full physics simulation in Gazebo (Koenig & Howard, 2004). The algorithm is evaluated in each environment with three radiation layouts generated randomly as described in Sect. 8.1.1.

For this set of experiments, and those in the following section, we use $p_{\min } = 0.99$, goal selection weights $\gamma _1 = 1.5$ and $\gamma _2 = 0.8$, batch evaluation size $N = 30$, gain threshold $\eta _{\texttt {gain}} = 0.1$, and volumetric gain parameters $\alpha _{\textit{unk}}=20.0$, $\alpha _{\textit{free}}=1.0$, $\alpha _{\textit{occ}}=0.0$. The GP kernel is an RBF with variance 1.0 and lengthscale ${2.0}\,\hbox {m}$. The GP observation noise $\sigma ^2 = 0.0009$, corresponding to $\sigma _\% \approx 3\%$ (Eq. 17). The robot’s initial position is randomly sampled from a set of 4 waypoints.

We evaluate our algorithm against two baselines. The first is a threshold-based algorithm which we denote ThresholdBasedExplorer (TB), which selects exploration goals using the same scoring function as SafeEstMDP-Map, but does so without an explicit hazard model. Instead, the safety function $\phi$ for ThresholdBasedExplorer treats states as safe to visit simply if the hazard value is no more than some safety limit *L*, or unsafe otherwise. It then uses a threshold fraction $\varphi \in (0, 1)$ of the safety limit to prevent it from entering unsafe states. If the robot reaches a state at which the radiation level is more than ${\varphi }L$ on the way to its goal, it marks all adjacent *unvisited* states as unsafe, and selects a new goal from the remaining safe states, thereby turning back from the path to the previous goal. The second baseline, MeanPredictionExplorer (MP), again uses the same goal selection process as SafeEstMDP-Map, except with a limited version of the full EstMDP. Rather than weighting the EstMDP intervals based on the GP posterior at each state, this baseline instead assigns all probability mass to the posterior mean value at each state. This baseline therefore classifies states as safe or unsafe based purely on the GP predictive mean, with no consideration of predictive uncertainty. We evaluate SafeEstMDP-Map against MeanPredictionExplorer and ThresholdBasedExplorer with $\varphi =0.3$ and $\varphi =0.8$, simulating each configuration for 30 exploration runs.

**Fig. 15:**
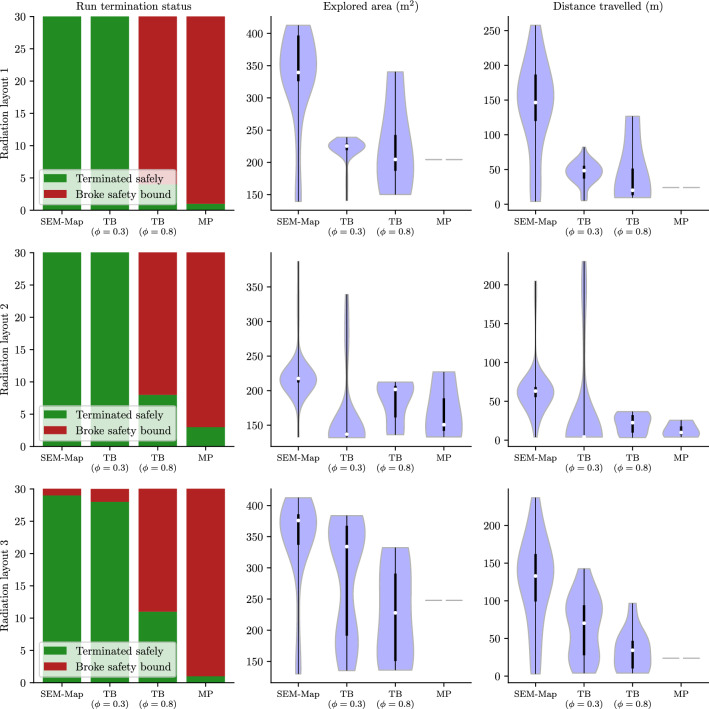
Experiments for SafeEstMDP-Map in the reactor room domain. Each algorithm is tested on three randomly generated radiation layouts, with each configuration repeated 30 times. Left: number of runs (out of 30) terminated successfully: middle: violin plots summarising the statistics over area explored; right: violin plots summarising the statistics over of distance travelled (Color figure online)

**Fig. 16:**
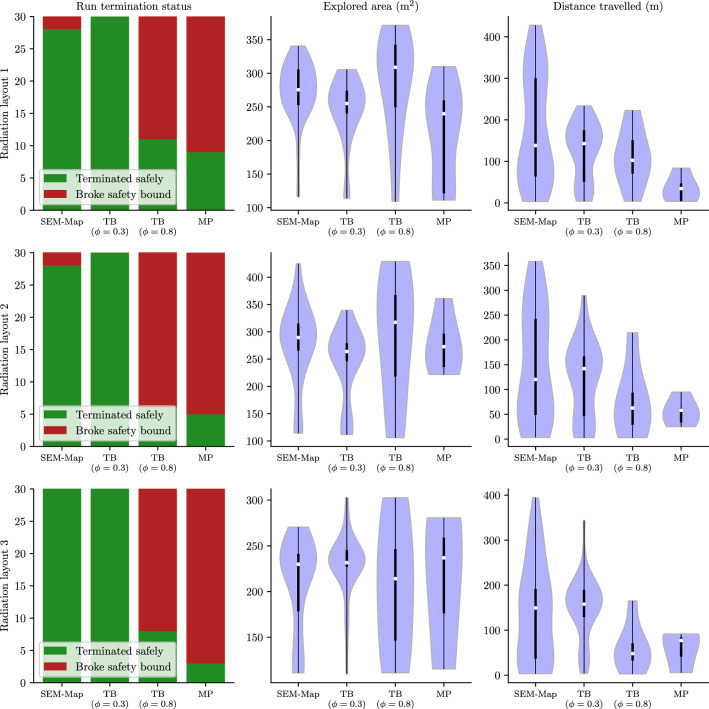
Experiments for SafeEstMDP-Map in the research mine domain for different radiation layouts. Each algorithm is tested on three randomly generated radiation layouts, with each configuration repeated 30 times. Left: number of runs (out of 30) terminated successfully: middle: violin plots summarising the statistics over area explored; right: violin plots summarising the statistics over of distance travelled (Color figure online)

The plots in Figs. 15 and 16 show that SafeEstMDP-Map is able to explore more effectively than the baselines across the variety of simulated configurations. The mean-prediction approach frequently results in unsafe termination, since without the full EstMDP model the safe reachability check is much less accurate, incorrectly classifying many unsafe states as safe. Of the threshold-based baselines, the more conservative one ($\varphi = 0.3$) is able to remain safe on almost every run. However, its exploration coverage on many of them is poorer than SafeEstMDP-Map’s, as it will tend to turn back as soon as its radiation reading begins to increase, whereas SafeEstMDP-Map’s radiation model allows it in some cases to continue forwards cautiously. On the other hand, the more aggressive baseline ($\varphi = 0.8$) explores more area, at the cost of far more runs terminating unsafely due to violating the safety specification.

SafeEstMDP-Map is able to use its GP model to explore more effectively, pushing forwards when an unexplored area is likely to be safe, and remaining cautious when it is believed unsafe. It also scales well with map size, as the EstMDP model is usually not much larger than the underlying navigation graph – even when the map is large, most waypoints will have been visited or be safe with probablility close to 1, so only the outer areas of the map will have significant uncertainty over radiation level. Consequently, the robot can evaluate dozens of goal choice waypoints per second during exploration, allowing for online planning with minimal delay, even in the research mine environment with maps of up to 150 waypoints. In some radiation layouts, SafeEstMDP-Map does still violate the safety constraint, although this is likely due to the GP model failing to accurately model the radiation field. This issue could be mitigated through further tuning or, more generally, applying domain knowledge in the design of the GP kernel.

## Deployment in a physical environment

We further validated our exploration algorithm by deploying it in a physical environment – the Corsham Research Mine – integrating it with a complete robotic hardware and software stack. Our experiments used simulated radiation sources, due to the health and safety difficulties of working with real radiation.

### Hardware and system configuration

**Fig. 17:**
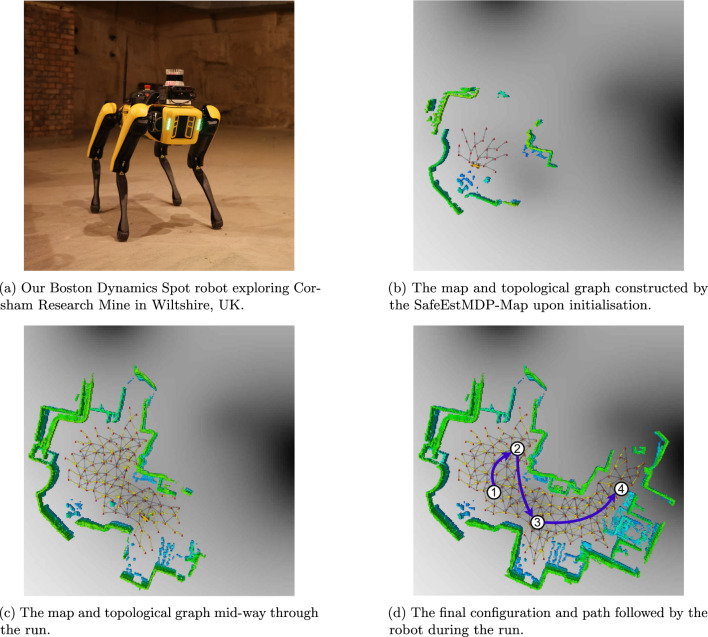
Progression of a single run of SafeEstMDP-Map in the Corsham Research Mine. **b**–**d** Show a 3D occupancy map representing the robot’s knowledge of the world around it (filtered for visual clarity), as well as a topological graph constructed online for planning and navigation. The grayscale background represents (ground truth) log radiation levels as a heatmap, with darker regions corresponding to higher levels

Our experiments were conducted on a Boston Dynamics Spot robot, shown with its sensor payload in Fig. 17a. The robot’s on-board computer was an Intel NUC with an Intel i7-8559U processor, a 256 GB SSD and 16 GB of RAM. For perception, the robot was equipped with an Ouster OS0-64 3D lidar with a full $360^\circ$ field of view around the robot. Localisation and mapping of the environment were provided by the VILENS SLAM system (Wisth et al., 2022), and interfaces between modules were through the robot operating system (ROS) Quigley et al. (2009).

The radiation simulation system (Sect. 8.1.1) must know the ground truth position of the robot and the radiation sources. In the fully simulated experiments, the ground-truth robot position is readily available. However, there was no external ground-truth source (e.g. a motion-capture system) available in Corsham Research Mine. To provide ground-truth robot positioning, we therefore ran a separate instance of the VILENS SLAM system in localisation mode (i.e. using a pre-built full coverage 3D map of the environment). Independently from the ground-truth localisation system, an *online* VILENS instance provided SLAM for each experiment run. The navigation graph construction component (Algorithm 4) used the coordinate system and OctoMap established by the online SLAM system.

### Deployment results

In order to illustrate the behaviour that can be obtained from the SafeEstMDP-Map in the real world, this section first steps through a successful run of the algorithm in the Corsham Research Mine. The output from this particular run is shown in Fig. 17. We then also comment on the exploration performance of the system across further deployments.

The robot started at the location shown in Fig. 17b, from which SafeEstMDP-Map constructed its initial topological map. The map at this point extended at most only a few metres from the robot’s position, in directions in which line-of-sight was unobstructed.

This area of the map had relatively low and uniform radiation levels, so the variation in the SafeEstMDP-Map scoring function (Eq. 15) was dominated by the volumetric gain term. The robot’s first plan was therefore to move up towards Location 2 in Fig. 17d, since the nodes in this direction were slightly more open than those to the right, and were therefore expected to provide more information for growing the map.

Upon reaching Location 2, the robot found the corridor blocked. Additionally, now that its map of this area was more complete, there was very little unobserved space nearby, so all nearby nodes had volumetric gain values below the required threshold to act as exploration candidates. Therefore, SafeEstMDP-Map turned back towards its other unexplored frontier at Location 3, where the nodes still had high exploration scores and were considered safe.

From Location 3, the robot was able to map the entire “room” located below it (Fig. 17c), thus there was no more unexplored space there and no need to explore further downwards. Instead, the robot moved to the right, incrementally extending its map towards Location 4 as shown in the figure. It is worth noting here that Fig. 17d does not show *every* goal selected by SafeEstMDP-Map during the run, but rather a simplified intuitive representation of the path followed – in reality, when moving into unexplored territory, the algorithm tends to plan only a few nodes ahead at a time, since far-away nodes cannot be known with high confidence to be safe.

As the robot approached Location 4, the radiation levels it measured began to increase, as indicated by the darker patch in Fig. 17d. As this happened, SafeEstMDP-Map became more cautious, tending to generate plans that only moved the robot forwards one node at a time, or had it visit additional nodes near the frontier rather than pushing aggressively into the unknown region. Ultimately, when the robot did reach Location 4, the unvisited nodes ahead of it were deemed unsafe based on the GP model. The unvisited nodes in the previously explored areas of the map, on the other hand, were still safe to visit, but their volumetric gain values were below the required threshold. There were therefore no more nodes that could serve as candidates for SafeEstMDP-Map, so the run terminated at Location 4.

This is overall a successful run of SafeEstMDP-Map, exhibiting the desired behaviours of the algorithm. It balanced exploitation of volumetric gain information with safety, exploring more aggressively when confident about safety, and becoming more cautious as safety became less certain.

In total, three full runs of SafeEstMDP-Map were conducted in the Corsham Research Mine using the radiation layout shown in Fig. 17. In all runs, the system was able to plan online and successfully explore the majority of the safely accessible area. With a total accessible area of approximately ${400}\,\hbox {m}^{2}$, the system achieved consistent results of $348.31\,\hbox {m}^{2}, 321.93\,\hbox {m}^{2}, 345.07\,\hbox {m}^{2}$ of the environment explored, without violating the safety specification.

## Conclusions

In this paper, we presented two algorithms, based on decision-making under uncertainty and GP modelling of unknown processes, for safe exploration and mapping of unknown environments. The first algorithm, SafeEstMDP-Process, assumes a known map and safely learns the distribution of an unknown hazard, whilst the second algorithm, SafeEstMDP-Map, drops the known map assumption and focuses on safely building an environment map. Both these algorithms rely on the EstMDP, a novel model which considers the uncertainty over a GP posterior of the underlying unknown process as part of its transition function. Our experiments show that our algorithms are able to safely explore in a two domains with a range of radiation configurations, and that the approach can be run in a real robot. In the future, we intend to extend the approach to goal-driven behaviour in unexplored regions, and introduce lookahead when considering where to explore, in contrast with the next-best-state selection approach presented in this paper.

## Figures and Tables

**Fig. 18:**
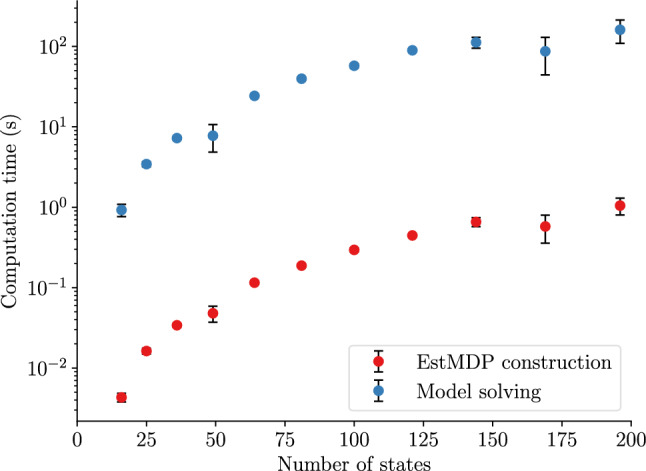
Scaling of computation time used for EstMDP construction (which includes all GP inference) and model solving versus number of states. Values are cumulative over complete runs of SafeEstMDP-Process on nuclear grid map domains of different sizes, using flat radiation layouts where all states are safely reachable. Each configuration was repeated 50 times

**Fig. 19:**
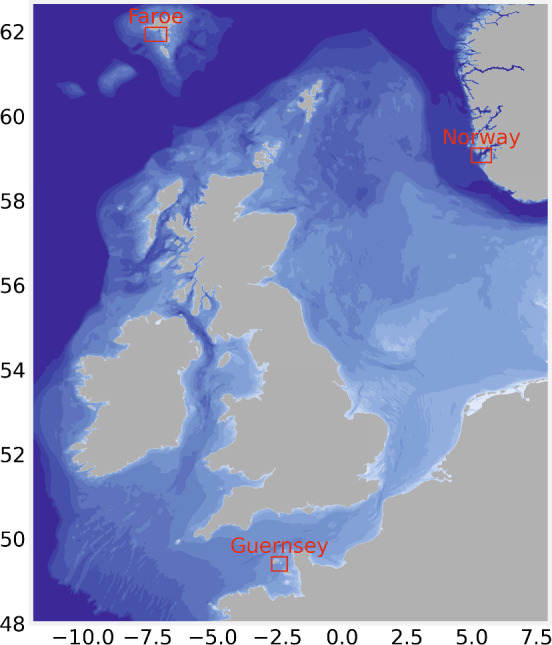
Location of the evaluation areas in the northwest European shelf. Blue colour levels indicate bathmetry depth

**Fig. 20:**
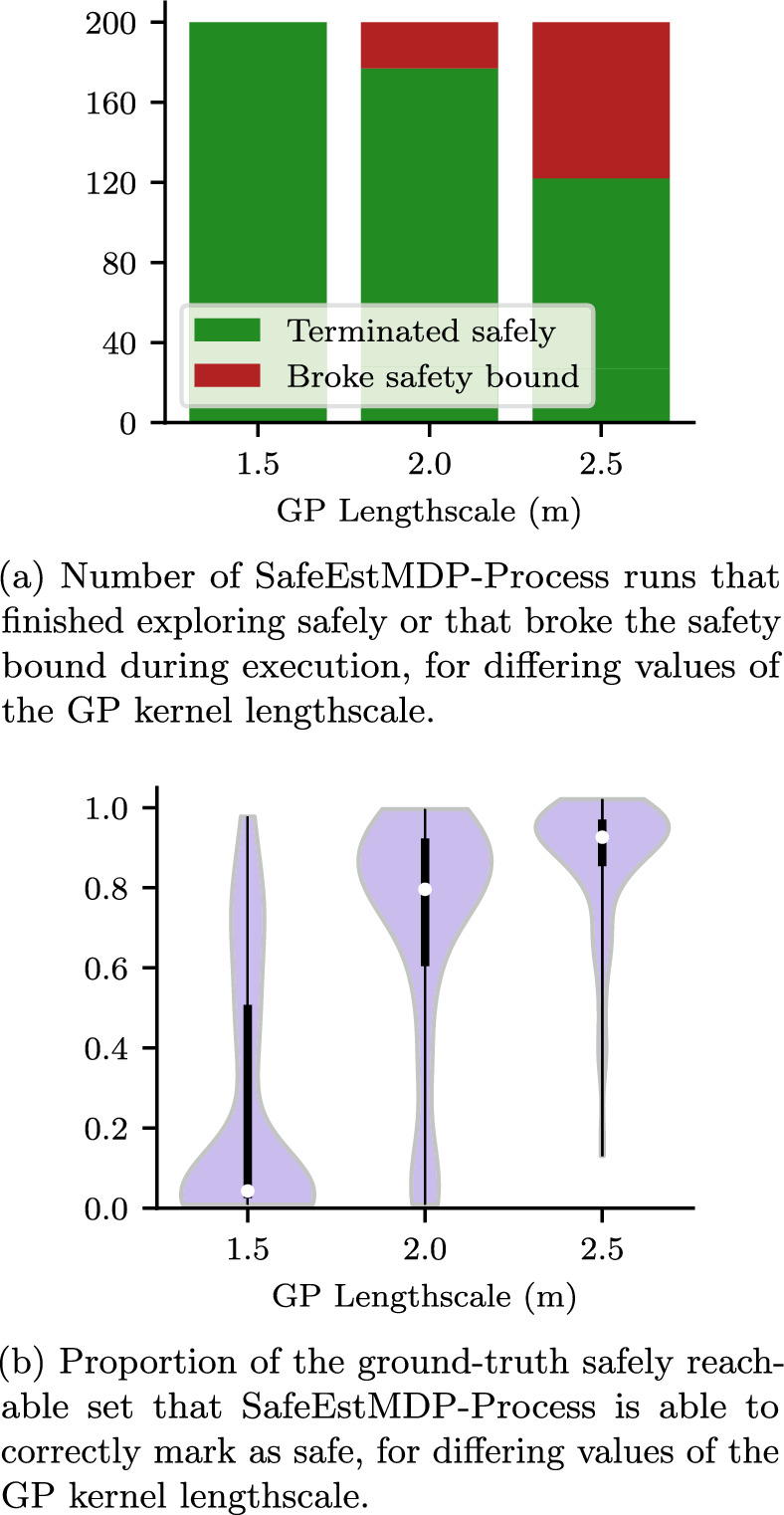
The effects of varying GP kernel lengthscale (x axis) on the behaviour of SafeEstMDP-Process, using a GP model *without* log-warping. Total 200 runs per GP lengthscale value (2 domains, 20 randomly generated radiation layouts, 5 repeats)

**Fig. 21:**
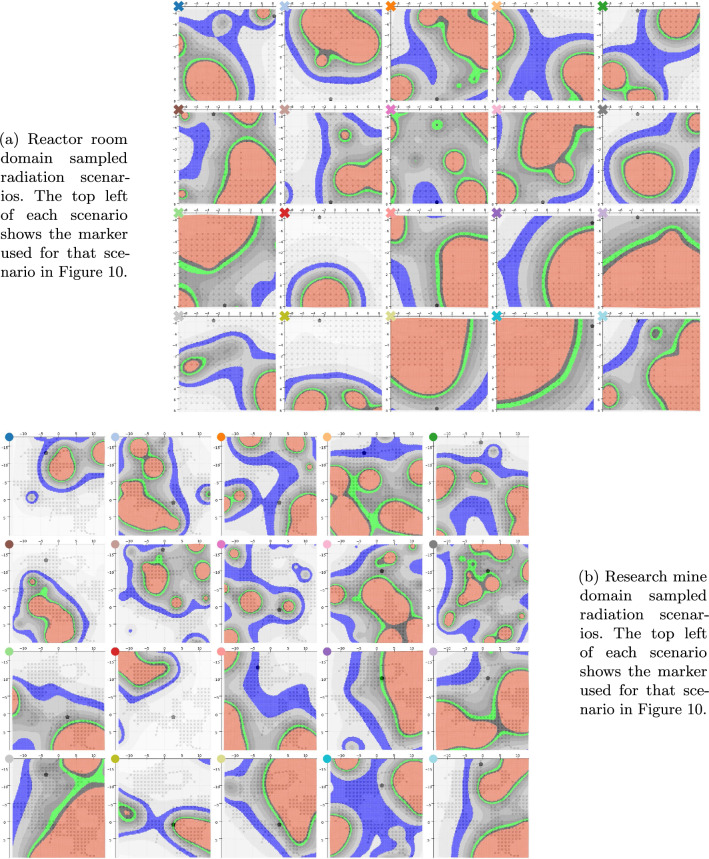
Randomly sampled radiation scenarios used in Sect. 8.2. The black hexagon shows the robot’s initial location. Contour levels are coloured blue to show a radiation level *rad* which is $30\% \le rad < 40\%$ of the safety bound, and green to show $80\% \le rad < 90\%$ of the safety bound. Red shows levels above the safety bound. The two rows of each figure were generated with the point source method, and the bottom two rows were generated with the Gaussian field method

## Appendix

### Computational complexity

In SafeEstMDP-Process and SafeEstMDP-Map, each goal selection step requires building and solving an EstMDP, which involve respectively (i) performing GP inference for each waypoint, and (ii) solving the resulting MDP using value iteration. Both of these problems have polynomial computational complexity with respect to the number of waypoints. In contrast, the POMDP planning methods mentioned in Sect. 2 are PSPACE-complete for finite horizons, and would not scale to problems of this size and larger. Figure 18 shows that, for the domain sizes considered in this work, the EstMDP solution step occupies significantly more computation time than the model construction step. As such, GP inference does not act as a computational bottleneck to the scalability of our approach.

### Autonomous underwater vehicle domain details

See Appendix Fig. 19.

For the AUV domain we implemented a policy cost check alongside the policy safety check, and abandoned the current goal when the cost of the policy to reach that goal from the current state was more than 2 times the expected cost-to-goal from that state as was calculated at goal selection time.

Due to the fact that (with the absence of current) the AUV’s motion takes it 2 cells per action, we also edited goal policy generation and execution to allow the policy executor to visit a goal state by passing through it (hence observing it) rather than solely by surfacing at the goal state.

The AUV dive depth was 10 m.

### GP comparison

See Appendix Fig. 20.

### Nuclear domain radiation scenarios

See Appendix Fig. 21.

## Footnotes

1 UK Met Office Marine Data Service. Available: https://www.metoffice.gov.uk/services/data/met-office-marine-data-service.
